# Patterns of prescription medicine dispensing before and during pregnancy in New Zealand, 2005–2015

**DOI:** 10.1371/journal.pone.0234153

**Published:** 2020-06-02

**Authors:** Sarah Donald, Katrina Sharples, David Barson, Simon Horsburgh, Lianne Parkin

**Affiliations:** 1 Pharmacoepidemiology Research Network, Dunedin, New Zealand; 2 Department of Preventive and Social Medicine, Dunedin School of Medicine, University of Otago, Dunedin, New Zealand; 3 Department of Mathematics and Statistics, University of Otago, Dunedin, New Zealand; 4 Department of Medicine, University of Otago, Dunedin, New Zealand; University of Oslo, NORWAY

## Abstract

**Objective:**

To describe prescription medicine dispensing before and during pregnancy in New Zealand, 2005–2015.

**Methods:**

Members of the New Zealand Pregnancy Cohort were linked with their dispensing records in a national database of prescription products dispensed from community pharmacies. We identified the proportion of pregnancies during which at least one prescription medicine was dispensed, the number of different medicines used and the most commonly dispensed medicine groups both during pregnancy and in the 270 days before conception. Dispensing during pregnancy was assessed by several maternal characteristics.

**Results:**

874,884 pregnancies were included. Over the study timeframe, the proportion of pregnancies exposed to a non-supplement prescription medicine increased from 38.5% to 67.2%. The mean number of different non-supplement medicines dispensed during pregnancy increased from 2.5 to 3.2. Dispensing during pregnancy was weakly associated with body mass index, smoking status and ethnicity. Pregnancy exposure was highest for Antibacterials (26.0%), Analgesics (16.7%) and Antinausea & Vertigo Agents (11.0%).

**Conclusions:**

From 2005–2015, both the proportion of exposed pregnancies and the number of different medicines dispensed to pregnant women in New Zealand increased.

## Introduction

Although many medicines lack evidence on their risk in pregnancy [1, 2] there is still substantial use of prescription medicines during pregnancy [3], with exposure increasing over recent decades [4–6]. Some recent estimates from Western countries show the proportions of pregnancies exposed to at least one medication ranged from 46%–93% [4, 5, 7–13]. Many pregnant women take multiple different medicines [10, 13–15]. Although patterns of use vary by country, systemic antibacterials, anti-emetics, gynaecological anti-infectives and antihistamines are commonly among the most dispensed medications during pregnancy [5, 8–10, 13–23].

Despite a deficit of safety information, the management of chronic or acute conditions during pregnancy may require continuation of an already prescribed medicine and/or the initiation of new therapies. A high proportion of pregnancies are unplanned [24], so that fetal exposure to medicines may occur in the early stages of organogenesis before the pregnancy is recognised. Awareness of medicine utilisation patterns allows the appropriateness of prescribing during pregnancy to be assessed and any concerning trends to be addressed. It may also highlight priority research areas.

There have been no comprehensive investigations of prescription medicine use during pregnancy in New Zealand. New Zealand has a publicly funded health care system, with free hospital and maternity care, as well as subsidised prescription medicines. Information on the use of these health services is held in national databases; these data have been used to construct a pregnancy cohort which can now be linked with dispensing records to investigate medicine use during pregnancy.

The aims of this study were to i) describe trends in prescription medicine dispensing before and during pregnancy in New Zealand from 2005–2015, and ii) examine prescription medicine dispensing during pregnancy by maternal characteristics.

## Methods

### Study cohort

This study was undertaken using the New Zealand Pregnancy Cohort, a national cohort containing 941,468 pregnancies over the period 2005–2015. The generation of this cohort has been described [25]. Briefly, records of pregnancies were identified in four New Zealand national health databases (covering hospital discharges, maternity care, laboratory tests (antenatal blood tests) and mortality records). Pregnancies with records in more than one database were matched using a unique patient identifier (an encrypted National Health Index (NHI)) and the pregnancy dates extracted or estimated from each database. Cohort members whose records lacked a last menstrual period (LMP) date or gestational age data (15%) had their LMP date estimated from an ICD-10-AM ‘duration of pregnancy’ code (O09) [26], which provided a range of earliest and latest possible gestational ages at the time of pregnancy end.

The pregnancy cohort includes all identified pregnancies of women who were 15–49 years of age at the start of pregnancy and had an LMP date between 1 January 2005 and 15 March 2015. In addition to virtually all deliveries of live and/or stillborn infants, the cohort also contains most other recognised pregnancies–those during which antenatal blood testing was undertaken and/or the woman received hospital care for a miscarriage, termination, or other early pregnancy loss. Not all cohort pregnancies have a known outcome; most of these pregnancies were identified by an early antenatal blood test only, and were presumed to be pregnancies that did not progress past the early stages. Maternal characteristics available for cohort members included maternal age, prioritised ethnicity, deprivation (New Zealand Index of Deprivation—NZDep) quintile, body mass index (BMI), smoking status and parity (primiparous vs. non-primiparous). Further description of these variables is available in S1 File.

For this study, only pregnancies with an LMP date from 15 September 2005 were included to ensure that 270 days of pre-conception dispensing data were available.

### Dispensing data

Dispensings of publicly funded medications from community pharmacies in New Zealand are recorded in the national Pharmaceutical Collection (PHARMS) [27]. PHARMS does not use the Anatomical Therapeutic Chemical (ATC) classification system but instead products are organised into therapeutic groups. In general, Level 1 therapeutic groups are organised by body system (e.g. Nervous System), with Level 2 therapeutic groups organised by functional group (e.g. Antidepressants) and Level 3 by drug class (e.g. Selective Serotonin Reuptake Inhibitors). Individual drugs (e.g. citalopram) have a unique chemical id number.

Using the NHI, records of dispensings occurring from 270 days prior to conception through to the end of pregnancy were linked with each pregnancy. Dispensings of medical devices, vaccines, food products and non-medicinal dermatological or compounding products were excluded (S1 Table). Dispensings of vitamins, minerals and folic acid were retained and categorised as supplements (S2 Table).

Exposure was defined as the dispensing of ≥1 prescription medicine (i.e. a filled prescription) during the time period of interest. Dispensings in eight time periods were considered: three consecutive 90-day pre-pregnancy periods (1–90, 91–180, and 181–270 days pre-conception), the entire 9-month pre-pregnancy period, each of the three pregnancy trimesters, and the entire pregnancy (S1 Fig). Conception was assumed to have occurred 14 days after the LMP date.

### Analyses

Changes in the proportion of pregnancies with ≥1 non-supplement dispensing by year of LMP, for each of the pre-pregnancy and pregnancy periods, were assessed. The denominator for Trimesters 2 and 3 was the number of pregnancies that had persisted to the start of the trimester. For any given pregnancy, we did not left censor the pre-pregnancy period for a previous pregnancy occurring within the previous 270 days. Sensitivity analyses were carried out to assess the impact of 1) using the earliest or latest estimated LMP dates for the 15% of the cohort with a range of LMP dates, and 2) left censoring those with a previous pregnancy in the pre-pregnancy period. The proportions of pregnancies in which ≥1 non-supplement medicine was dispensed were compared by pregnancy outcome (for Trimester 1), and by maternal characteristics. For pregnancies with at least one non-supplement dispensing, changes in the number of different medicines dispensed over time (year of LMP) were assessed, as well as differences by maternal characteristics and by pregnancy outcome (for Trimester 1).

Dispensings were examined to determine which medicine groups (Therapeutic Group Level 2) were associated with ≥1% of the pregnancies, in either the 270-day pre-pregnancy period or during pregnancy. Those therapeutic groups dispensed during ≥1% of pregnancies were examined for changes in proportions over time (in 3 time periods: 2005–2008, 2009–2011, 2012–2015).

Multiple imputation using chained equations (*m* = 40 imputations) was used to estimate missing maternal characteristic data. The imputation model included the year of LMP, all six maternal characteristics, three outcome variables and several auxiliary variables derived from the cohort members’ past dispensings and hospitalisations. Full details of the imputation model are available in S2 File.

A generalised estimating equation framework was used for analyses of proportions with ≥1 dispensing, to allow for clustering of more than one pregnancy within a mother. Proportions were compared using relative risk regression [28] with Poisson errors, robust standard errors and clustered by mother. Analyses of the number of medicines dispensed in those with ≥1 dispensing were modelled using negative binomial regression (adjusted for clustering by mother) to estimate the relative change in the mean number of different medicines used. Analyses were done by trimester to account for duration of pregnancy. All analyses were carried out using STATA v14.2.

### Ethics statement

Written ethics approval for this study was obtained from the Northern A Health and Disability Ethics Committee (16/NTA/76).

## Results

The analyses were based on 874,884 pregnancies to 468,480 women (see S3 Table for the number of pregnancies per cohort member). For each pregnancy, maternal characteristics at the time of the pregnancy and the pregnancy outcome are shown in Table 1. The relative proportions of pregnancy outcomes did not change substantially over the study period (S4 Table). All findings reported in this paper are based on the analyses undertaken using the earliest LMP dates, which did not vary substantially from those found using the latest LMP dates.

**Table 1:** Maternal characteristics at the time of pregnancy and pregnancy outcomes.

Maternal characteristic	Number of pregnancies (n = 874,884)	Proportion of pregnancies (%)
**Age group (years)**		
15–19	89,854	10.3
20–29	385,554	44.1
30–39	361,471	41.3
40–49	38,005	4.3
**Ethnicity (prioritised)**^a^		
European	376,770	43.1
Māori	230,484	26.3
Pacific	99,646	11.4
Asian	111,581	12.8
MELAA^b^	54,571	6.2
Other	514	0.1
Missing	1,318	0.2
**Deprivation (NZDep) quintile**		
1 (least deprived)	123,534	14.1
2	134,824	15.4
3	161,345	18.4
4	204,533	23.4
5 (most deprived)	249,777	28.5
Missing	871	0.1
**BMI category**		
Underweight (<18)	12,647	1.4
Healthy weight (18–<25)	207,660	23.7
Overweight (25–<30)	127,760	14.6
Obese (≥30)	106,943	12.2
Missing	419,874	48.0
**Smoking status**		
Non-smoker	398,687	45.6
Smoker	154,745	17.7
Missing	321,452	36.7
**Parity**		
Primiparous	207,328	23.7
Non-primiparous	475,579	54.4
Missing	191,977	21.9
**Pregnancy Outcome**		
Live birth(s) only	583,127	66.7
Stillbirth(s) only	1,768	0.2
Live and stillbirth(s)	92	0.0
Miscarriage	46,184	5.3
Termination	80,594	9.2
Other early pregnancy loss^c^	4,131	0.5
Undetermined	158,988	18.2

a Ethnicity prioritised according to Statistics New Zealand Level 1 ethnic groups

b Middle Eastern/Latin American/African

c Extrauterine pregnancy, non-viable products of conception

Overall, 48.9% of pregnancies were missing data for at least one maternal characteristic.

Overall, a prescription medicine or supplement was dispensed at least once during 69.4% of pregnancies. However, the proportion of exposed pregnancies increased over time, rising from 43.9% in 2005 to 82.3% in 2015. This increase was accompanied by a corresponding, though less pronounced, increase in exposure in the 270-day pre-pregnancy period, from 47.8% to 71.8%.

**Fig 1:**
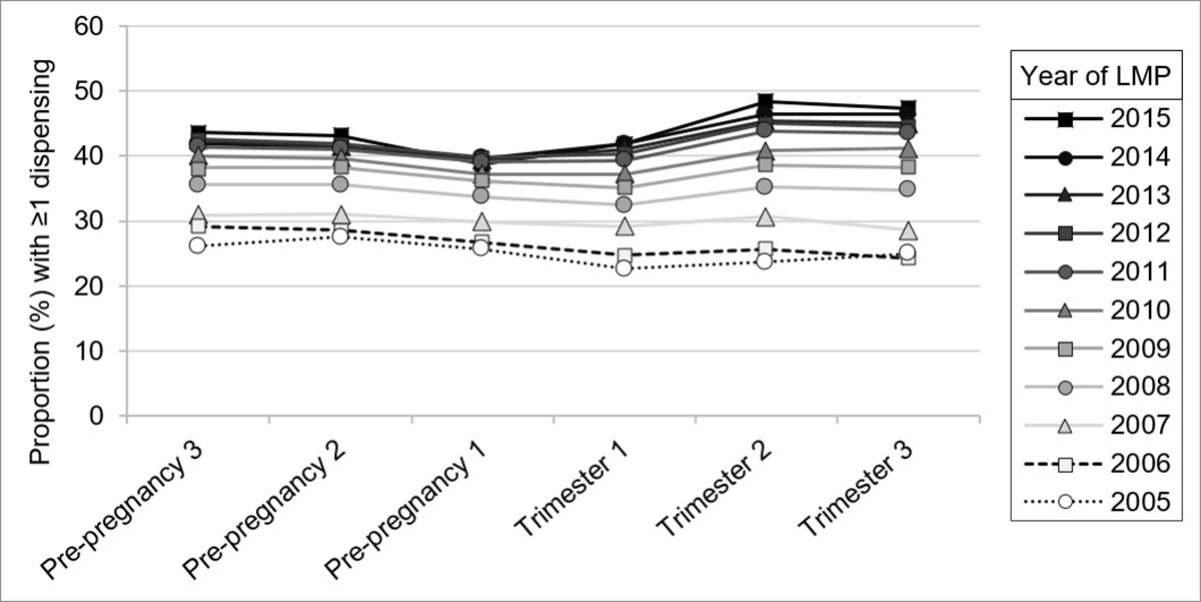
The proportion of pregnancies with at least one dispensing of a non-supplement prescription medicine. Pre-pregnancy 3 = 181–270 days pre-conception, Pre-pregnancy 2 = 91–180 days pre-conception, Pre-pregnancy 1 = 1–90 days pre-conception. The denominators for Trimesters 2 and 3 include only those pregnancies that persisted to the start of the respective trimester.

After excluding supplements, ≥1 dispensing occurred during 57.8% of all pregnancies, and in 64.6% of pregnancies ending in the delivery of a live and/or stillborn infant. An increase in the exposed proportion over time was evident in all pre-pregnancy and pregnancy periods (Fig 1). For the whole cohort, exposure during pregnancy increased from 38.5% in 2005 to 67.2% in 2015 (RR 1.75 [1.72–1.78]). Although the exposed proportions in the 90-days prior to pregnancy and in Trimester 1 were very similar overall (35.3% and 35.5% respectively), exposure was slightly higher in Pre-pregnancy 1 until 2010, after which time exposure in Trimester 1 became slightly higher. For pregnancies ending in delivery, the exposed proportions for the individual pre-pregnancy and pregnancy periods were very similar to those of the whole cohort (S5 Table). Results for the sensitivity analyses of the exposed proportions using the earliest and latest LMP dates are shown in S6 Table. Proportions with pre-pregnancy exposure to ≥1 non-supplement medicine did not change substantially when pregnancies with a previous pregnancy in the 270-day pre-pregnancy period were excluded from the analyses (S7 Table).

**Fig 2:**
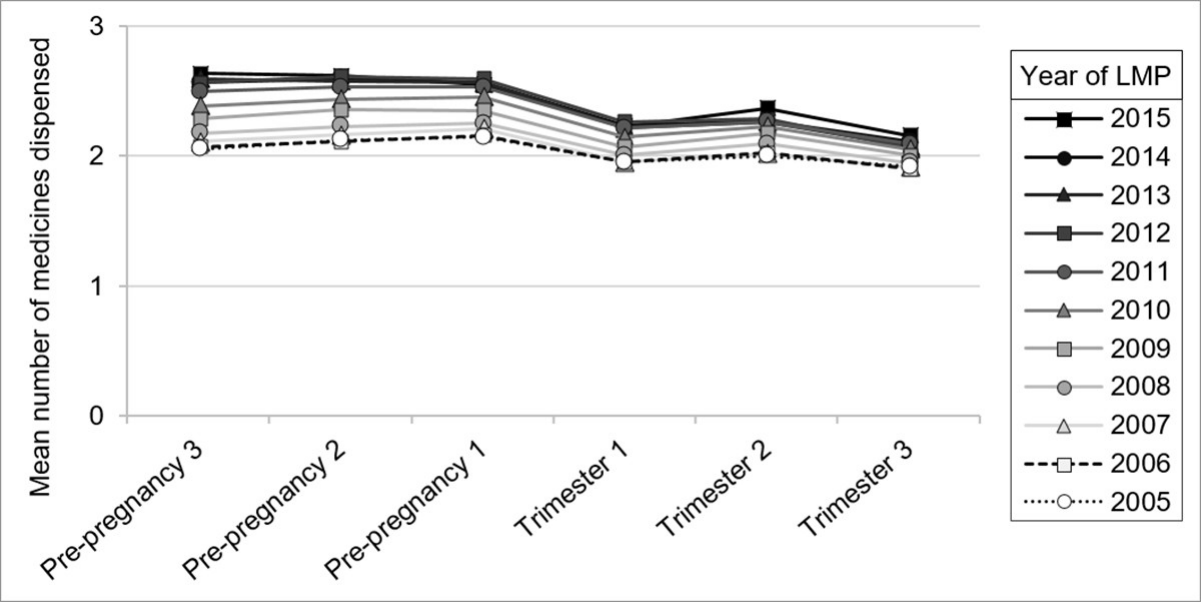
The mean number of different non-supplement prescription medicines dispensed to cohort members with at least one dispensing before and during pregnancy, 2005–2015. Pre-pregnancy 3 = 181–270 days pre-conception, Pre-pregnancy 2 = 91–180 days pre-conception, Pre-pregnancy 1 = 1–90 days pre-conception.

The proportion of pregnancies in which multiple non-supplement medicines were dispensed rose over the study period, from 14.6% to 25.3% for 2–3 different medicines, from 6.9% to 19.1% for 4–7 medicines, and from 1.2% to 4.3% for ≥8 medicines. Considering only the pregnancies with ≥1 non-supplement dispensing, the mean number of different medicines dispensed for the cohort overall was 2.9, increasing from 2.5 in 2005 to 3.2 in 2015 (RR 1.49 [1.44–1.53]). The mean number of medicines dispensed during pregnancies that ended in delivery was 3.1. Over the study timeframe, the mean number of different medicines dispensed increased in all pre-pregnancy and pregnancy periods (Fig 2). The mean number of different medicines dispensed in the 270 days pre-pregnancy also increased over the study timeframe, from 2.8 to 3.9 (RR 1.57 [1.52–1.61]).

During Trimester 1, exposure to ≥1 non-supplement was slightly higher in pregnancies that ended early compared to those ending in a delivery (38.8% vs 34.6%), with a similar mean number of medicines dispensed in both groups (2.3 vs 2.1) (Table 2).

**Table 2:** Non-supplement dispensing in Trimester 1, by grouped pregnancy outcome.

Pregnancy outcome	Trimester 1					
	Proportion with ≥1 dispensing			Number of medicines dispensed		
	%	aRR^a^	95% CI	Mean^b^	aRR^a^	95% CI
Deliveries^c^	34.6	1.00	reference	2.1	1.00	reference
Non-deliveries^d^	38.8	1.12	[1.11–1.13]	2.3	1.14	[1.13–1.16]
Undetermined	34.5	1.00	[0.99–1.01]	2.1	1.04	[1.03–1.06]

^a^ Adjusted for year of LMP and clustering by mother

^b^ Mean only includes pregnancies with at least one dispensing

^c^ Includes deliveries of live and/or stillborn infants

^d^ Includes terminations, miscarriages, other early pregnancy losses

After adjusting for the other maternal characteristics, year of LMP and clustering by mother, the likelihood of being dispensed a non-supplement medicine at least once during a pregnancy trimester was not strongly associated with the maternal characteristics investigated (Table 3). In all trimesters, a dispensing was less likely for Asian women versus European women, whereas Māori women were marginally less likely to have had ≥1 dispensing in Trimester 1 (aRR 0.92 [0.92–0.93]), and Trimester 3 exposure was slightly lower in Pacific women (aRR 0.93 [0.92–0.94]. A dispensing was slightly more likely in all trimesters if a woman was obese versus a healthy weight, or was more deprived. Exposure was higher in smokers in Trimester 1 (aRR 1.09 [1.08–1.09]) and Trimester 3 (aRR1.08 [1.07–1.09]), and in non-primiparous women in Trimester 3 (aRR1.11 [1.10–1.12]).

**Table 3:** The proportion of pregnancies with ≥1 dispensing of a non-supplement prescription medicine during each of the pregnancy trimesters, by maternal characteristics.

Maternal characteristic	Proportion with at least one dispensing of a non-supplement								
	Trimester 1			Trimester 2			Trimester 3		
	%^a^	aRR^b^	[95% CI]	%^a^^c^	aRR^b^	[95% CI]	%^a^^c^	aRR^b^	[95% CI]
**Age group (years)**	** **			** **			** **		
15–19	36.5	1.06	[1.05–1.07]	40.7	1.07	[1.06–1.08]	39.8	1.12	[1.10–1.13]
20–29	36.0	1.00	reference	39.2	1.00	reference	37.5	1.00	reference
30–39	33.8	0.95	[0.95–0.96]	37.4	0.98	[0.97–0.99]	37.9	1.03	[1.02–1.04]
40–49	37.2	1.03	[1.01–1.05]	39.7	1.01	[0.99–1.02]	42.9	1.12	[1.10–1.14]
**Ethnicity (prioritised)**^**d**^	** **			** **			** **		
European	35.2	1.00	reference	37.8	1.00	reference	38.0	1.00	reference
Māori	36.0	0.92	[0.92–0.93]	40.9	1.00	[0.99–1.01]	39.8	0.95	[0.94–0.96]
Pacific	37.2	0.95	[0.94–0.96]	42.1	1.00	[0.99–1.01]	39.6	0.93	[0.92–0.94]
Asian	31.9	0.91	[0.90–0.92]	33.7	0.89	[0.88–0.90]	33.4	0.89	[0.88–0.90]
MELAA^e^	35.1	1.04	[1.02–1.05]	37.3	1.03	[1.01–1.04]	37.0	1.01	[1.00–1.03]
Other	31.9	0.91	[0.79–1.05]	30.8	0.82	[0.70–0.95]	37.7	0.99	[0.86–1.15]
**NZDep quintile**	** **			** **			** **		
1 (least deprived)	32.5	1.00	reference	35.2	1.00	reference	35.3	1.00	reference
2	33.5	1.02	[1.01–1.03]	36.4	1.02	[1.01–1.03]	36.2	1.01	[1.00–1.03]
3	34.4	1.04	[1.03–1.05]	37.5	1.04	[1.03–1.06]	37.3	1.04	[1.03–1.05]
4	36.1	1.08	[1.07–1.09]	38.9	1.07	[1.06–1.08]	38.7	1.07	[1.06–1.08]
5 (most deprived)	37.2	1.09	[1.08–1.10]	42.0	1.11	[1.10–1.13]	40.4	1.08	[1.07–1.09]
**BMI category**	** **			** **			** **		
Underweight (<18)	31.2	0.98	[0.95–1.00]	33.2	0.97	[0.95–0.99]	31.4	0.94	[0.92–0.97]
Healthy weight (18 - <25)	32.3	1.00	reference	34.9	1.00	reference	34.1	1.00	reference
Overweight (25 - <30)	36.2	1.10	[1.09–1.11]	39.7	1.10	[1.09–1.11]	39.2	1.12	[1.11–1.13]
Obese (≥30)	40.1	1.19	[1.18–1.20]	45.0	1.22	[1.21–1.23]	44.9	1.26	[1.25–1.27]
**Smoking status**	** **			** **			** **		
Non-smoker	34.4	1.00	reference	38.1	1.00	reference	37.4	1.00	reference
Smoker	37.5	1.09	[1.08–1.09]	40.0	1.02	[1.01–1.03]	40.4	1.08	[1.07–1.09]
**Parity**	** **			** **			** **		
Primiparous	33.4	1.00	reference	36.8	1.00	reference	35.0	1.00	reference
Non-primiparous	36.1	1.07	[1.06–1.08]	39.6	1.05	[1.04–1.06]	39.9	1.11	[1.10–1.12]

^**a**^ Proportions include imputed data (m = 40)

^**b**^ Adjusted for all other factors in the table, year of earliest LMP, and clustering by mother

^c^ Denominator includes only those pregancies that persisted to the start of the trimester

^**d**^ Ethnicity prioritised according to Statistics New Zealand Level 1 ethnic groups

^**e**^ Middle Eastern/Latin American/African

The mean number of different non-supplement medicines dispensed during the pregnancy trimesters did not vary substantially by the different maternal characteristics; obesity, smoking, and the oldest age group were weakly associated with the dispensing of more medicines (Table 4). The relative risks for associations with maternal characteristics found in the complete case analyses were broadly similar to those reported above (S8 Table).

**Table 4:** The number of different non-supplement prescription medicines dispensed during each of the pregnancy trimesters, by maternal characteristics.

Maternal characteristic	Number of different non-supplements dispensed								
	Trimester 1			Trimester 2			Trimester 3		
	Mean^a^	aRR^b^	[95% CI]	Mean^a^	aRR^b^	[95% CI]	Mean^a^	aRR^b^	[95% CI]
**Age group (years)**	** **			** **			** **		
15–19	2.1	1.01	[1.00–1.02]	2.1	0.99	[0.98–1.00]	2.0	1.01	[1.00–1.02]
20–29	2.2	1.00	reference	2.2	1.00	reference	2.0	1.00	reference
30–39	2.1	0.98	[0.98–0.99]	2.2	1.01	[1.00–1.01]	2.1	1.02	[1.02–1.03]
40–49	2.2	1.04	[1.02–1.05]	2.4	1.07	[1.05–1.08]	2.2	1.08	[1.06–1.10]
**Ethnicity (prioritised)**^**c**^	** **			** **			** **		
European	2.1	1.00	reference	2.2	1.00	reference	2.0	1.00	reference
Māori	2.2	0.98	[0.97–0.99]	2.2	1.00	[0.99–1.01]	2.1	0.97	[0.96–0.98]
Pacific	2.2	1.01	[1.00–1.02]	2.3	1.00	[0.99–1.01]	2.0	0.95	[0.94–0.96]
Asian	2.1	1.04	[1.03–1.05]	2.1	1.01	[1.00–1.02]	2.0	0.99	[0.98–1.00]
MELAA^d^	2.1	1.03	[1.02–1.05]	2.2	1.03	[1.01–1.04]	2.0	1.00	[0.99–1.02]
Other	2.1	0.99	[0.87–1.14]	2.2	1.01	[0.91–1.13]	2.1	1.01	[0.89–1.15]
**NZDep quintile**	** **			** **			** **		
1 (least deprived)	2.0	1.00	reference	2.1	1.00	reference	2.0	1.00	reference
2	2.0	1.01	[0.99–1.02]	2.1	1.01	[1.00–1.02]	2.0	1.01	[1.00–1.02]
3	2.1	1.03	[1.02–1.04]	2.2	1.02	[1.01–1.04]	2.0	1.02	[1.01–1.03]
4	2.2	1.05	[1.04–1.06]	2.2	1.04	[1.02–1.05]	2.1	1.04	[1.03–1.05]
5 (most deprived)	2.2	1.05	[1.04–1.06]	2.3	1.05	[1.04–1.06]	2.1	1.03	[1.02–1.04]
**BMI category**	** **			** **			** **		
Underweight (<18)	2.0	0.99	[0.97–1.01]	2.0	0.98	[0.96–1.00]	1.9	1.00	[0.97–1.02]
Healthy weight (18 - <25)	2.0	1.00	reference	2.0	1.00	reference	1.9	1.00	reference
Overweight (25 - <30)	2.1	1.06	[1.05–1.07]	2.2	1.06	[1.06–1.07]	2.0	1.07	[1.06–1.07]
Obese (≥30)	2.3	1.15	[1.14–1.16]	2.4	1.17	[1.16–1.18]	2.2	1.16	[1.15–1.17]
**Smoking status**	** **			** **			** **		
Non-smoker	2.1	1.00	reference	2.2	1.00	reference	2.0	1.00	reference
Smoker	2.2	1.08	[1.07–1.09]	2.2	1.03	[1.02–1.04]	2.1	1.07	[1.06–1.08]
**Parity**	** **			** **			** **		
Primiparous	2.1	1.00	reference	2.1	1.00	reference	1.9	1.00	reference
Non-primiparous	2.2	1.03	[1.02–1.04]	2.2	1.02	[1.01–1.03]	2.1	1.05	[1.04–1.06]

^a^ Means include imputed data (m = 40), and only include pregnancies with at least one dispensing during the specified

^b^ Adjusted for all other factors in the table, year of earliest LMP, and clustering by mother

^c^ Ethnicity prioritised according to Statistics New Zealand Level 1 ethnic groups

^d^ Middle Eastern/Latin American/African

Therapeutic groups dispensed during at least 1% of pregnancies are shown in Table 5 (relative risks and 95% confidence intervals for trends during pregnancy and for trends over time are available in S9 and S10 Tables respectively). All therapeutic groups dispensed to at least one cohort member before or during pregnancy are listed in S11 Table. Overall, Minerals (36.9%) and Antianaemics (27.8%) were dispensed in the highest proportion of pregnancies. Practically all of the pre-pregnancy and pregnancy Antianaemics dispensings were folic acid. Antibacterials (26.0%), Analgesics (16.7%) and Antinausea & Vertigo Agents (11.0%) were the most commonly dispensed non-supplements during pregnancy. Dispensing of therapeutic groups during Trimester 1 by pregnancy outcome are shown in S12 Table.

Non-supplement therapeutic groups in Table 5 that were dispensed more commonly during Trimester 1 than in the 30 days prior to pregnancy included the Antinausea & Vertigo Agents, Gynaecological Anti-infectives, Laxatives, Urinary Tract Infections, Antacids & Antiflatulants, Treatments for Substance Dependence, and Antithrombotic Agents.

**Table 5:** Proportion of pregnancies with at least one dispensing from the listed therapeutic groups during the pre-pregnancy and pregnancy periods.

Therapeutic group^a^	Proportion (%) of pregnancies with ≥1 dispensing in specified period									
	Pre-pregnancy 3^b^	Pre-pregnancy 2^c^	Pre-pregnancy 1^d^	Trimester 1	Trimester 2	Trimester 3	Whole pregnancy			
	All years						2005–08	2009–11	2012–15	All years
Minerals	3.7	3.5	3.0	18.7	20.1	32.1	14.6	39.6	59.8	36.9
Antianaemics	2.6	3.6	4.6	25.5	4.0	0.8	15.5	29.1	40.7	27.8
Antibacterials	13.6	14.0	13.7	11.0	14.4	13.1	21.4	28.3	28.9	26.0
Analgesics	7.8	8.1	7.8	6.7	9.6	7.9	10.8	18.7	21.6	16.7
Antinausea & Vertigo Agents	1.7	1.7	1.5	8.0	4.8	1.5	6.7	11.3	15.6	11.0
Gynaecological Anti-infectives	1.8	1.8	1.7	2.4	4.7	5.5	5.8	9.5	11.0	8.6
Corticosteroids Topical	3.6	3.6	3.5	3.1	4.2	3.4	6.2	8.4	8.4	7.6
Beta-Adrenoceptor Agonists	3.8	3.8	3.7	3.4	4.0	3.7	6.0	6.8	6.3	6.3
Vitamins	1.1	1.1	1.0	2.3	2.2	3.7	2.3	6.4	9.2	5.8
Laxatives	1.1	1.0	0.9	1.5	2.1	2.4	2.3	4.5	6.5	4.4
Urinary Tract Infections	1.2	1.2	1.3	1.7	2.3	1.7	3.3	4.7	5.2	4.3
Antidepressants	4.4	4.6	4.5	3.1	2.3	2.2	3.5	4.4	4.7	4.2
Antitrichomonal Agents	1.7	1.8	1.7	1.5	2.2	1.4	2.8	4.4	5.1	4.0
Antihistamines	2.8	2.9	2.9	1.9	1.8	1.5	2.7	4.3	5.2	4.0
Antiulcerants	1.4	1.5	1.4	1.2	1.6	2.7	2.1	3.6	5.4	3.6
Non-Steroidal Anti-Inflammatory Drugs	5.5	5.8	5.6	2.2	1.1	0.7	2.2	3.8	4.8	3.5
Inhaled Corticosteroids	2.0	2.0	1.9	1.7	2.0	1.8	3.6	3.3	2.5	3.2
Local preparations for Anal & Rectal Disorders	0.7	0.6	0.5	0.4	1.1	2.9	1.8	3.3	4.3	3.1
Antacids & Antiflatulants	0.2	0.2	0.2	0.7	1.5	2.2	2.2	3.9	3.1	3.1
Nasal Preparations	1.4	1.5	1.5	1.3	1.7	1.1	2.2	3.3	3.5	2.9
Contraceptives—Hormonal	8.6	7.2	4.5	1.9	1.2	0.1	2.5	2.9	3.2	2.9
Antifungals Topical	0.7	0.6	0.6	0.6	1.2	1.4	1.7	2.7	2.7	2.3
Diabetes	0.7	0.7	0.8	0.7	1.0	2.6	1.3	2.3	3.3	2.3
Corticosteroids & Related Agents for Systemic Use	1.3	1.4	1.4	0.8	1.0	0.9	1.5	2.2	2.3	2.0
Eye Preparations	1.1	1.2	1.2	0.8	0.9	0.7	1.6	2.1	2.2	1.9
Antibacterials Topical	0.9	0.9	0.9	0.7	0.9	0.7	1.3	2.1	2.0	1.8
Treatments for Substance Dependence	0.6	0.6	0.7	0.8	0.9	0.5	0.5	2.3	2.3	1.6
Antithrombotic Agents	0.2	0.2	0.2	0.8	1.4	0.8	0.6	1.5	3.0	1.6
Inhaled Long-acting Beta-adrenoceptor Agonists	1.1	1.1	1.2	1.1	1.1	1.1	1.1	1.6	2.0	1.5
Sedatives and Hypnotics	1.1	1.1	1.1	0.6	0.4	0.5	0.8	1.2	1.2	1.1
Thyroid & Antithyroid Agents	0.7	0.7	0.7	0.7	0.8	0.8	0.6	1.0	1.5	1.0

a PHARMS Level 2 therapeutic groups

b 181–270 days pre-conception

c 91–180 days pre-conception

d 1–90 days pre-conception

This table contains all therapeutic groups that were dispensed during at least 1% of the cohort pregnancies

Antibacterial dispensings were fairly stable throughout pre-pregnancy and pregnancy. Rising use throughout pregnancy was seen for Gynaecological Anti-infectives, Antiulcerants, Antacids & Antiflatulants, Topical Antifungals, and Diabetes. Conversely, dispensings of Antianaemics, Antinausea & Vertigo Agents, Antidepressants, Non-steroidal Anti-inflammatory Drugs, and Hormonal Contraceptives dropped throughout pregnancy.

For all therapeutic groups in Table 5 except Inhaled Corticosteroids, the proportion exposed during pregnancy increased over time. Compared to the earliest years of the study, the non-supplement groups with the highest absolute increase in the proportion of pregnancies with ≥1 dispensing by the end of the study were the Analgesics (+10.8%), Antinausea and Vertigo Agents (+8.9%) and Antibacterials (+7.5%). In terms of relative increase, the top non-supplement groups were the Antithrombotic Agents (RR 5.40 [5.12–5.70]), Treatments for Substance Dependence (RR 4.22 [4.00–4.45]), and Laxatives (RR 2.76 [2.69–2.84]).

## Discussion

We found that the proportion of pregnancies in which at least one non-supplement prescription medicine was dispensed increased substantially between 2005 and 2015, and the number of different medicines dispensed also rose. Exposure in early pregnancy was similar in all pregnancies regardless of outcome, but was higher during the whole pregnancy for those that progressed to a delivery. This discrepancy is likely attributable to the longer duration of such pregnancies. During pregnancy, dispensing did not vary substantially by the maternal characteristics; the strongest positive associations with higher exposure was in obese women.

This study is the first to describe prescription medicine dispensing patterns in pregnant women in New Zealand. A key strength is the inclusion of over 878,000 pregnancies–representing almost all of the live and stillbirths nationally between 2005 and 2015, as well as many of the pregnancies which did not progress past early pregnancy. Linkage of these pregnancies with national dispensing data from community pharmacies has provided a national picture of prescription medicine dispensing in pregnancy.

There were some limitations to this study which require further consideration. In New Zealand the use of folic acid, iodine and iron are recommended during pregnancy [29, 30] and these products are available both on prescription and over-the-counter. Despite evidence from this study showing that the proportion of pregnancies in which Minerals, Vitamins and Antianaemics (mostly folic acid) were dispensed increased considerably, the lack of information on patterns of over-the-counter purchases of these supplements limited our ability to draw meaningful conclusions regarding overall exposure patterns. However, our key focus was the use of non-supplement prescription medicines.

Our study used dispensing data, which does not necessarily equate to consumption, so could have overestimated medicines actually used in pregnancy. Pregnant women often overestimate the teratogenic risk of prescription medicines, which can reduce adherence during pregnancy [31–34]. However, several studies have shown high levels of concordance between self-reported medicine use and medical records for medicines used to treat chronic conditions during pregnancy, with somewhat lower concordance for medicines used intermittently or for short durations [35–39]. It is important to note that concordance may be affected not only by non-consumption but also by incorrect self-report (e.g. poor recall for short-term medicines [35, 36], or non-disclosure of socially stigmatised medicines [35] and medicines viewed as innocuous [40]). Dispensing claims databases are regarded as a valid data source for pregnancy pharmacoepidemiology studies [41, 42].

Although the exposure definition used in this study (≥1 dispensing) was fairly simple, it was appropriate for the study aims, which were to provide a general overview of prescription medicine dispensing in pregnancy and to describe changes over time. In future studies which aim to explore potential relationships between the use of specific medicines in pregnancy and health outcomes, it will be possible to employ more sophisticated exposure definitions which take dose and duration of use into account. We did not censor the pre-pregnancy period if there was a previous pregnancy within the 270 days examined, however our sensitivity analyses demonstrated that it would have made very little difference to our findings had we done so.

PHARMS does not include information about medicines dispensed in hospital, so exposure may have been underestimated if women were admitted to hospital during pregnancy. PHARMS records are compiled from reimbursement claims from community pharmacies, and the data quality is dependent upon the information submitted in the claims. The proportion of dispensing records (for all patients of all ages) without an NHI was 13.4% in 2005, 7.8% in 2006, and <5% by 2008. While a small part of the increase in dispensing seen in this study could be attributable to the improved NHI recording over time, the increases in dispensing were much higher than the improvement in NHI recording and most of the increase seen is probably due to real changes in dispensing patterns. Additionally, because exposure was simply defined as ≥1 dispensing, it is possible that while some dispensings to a particular woman were lacking an NHI, others did include an NHI, minimising exposure misclassification.

An appreciable proportion of records were missing data for BMI, smoking status and parity. We used multiple imputation to fill in the missing data, which should produce estimates with reduced bias compared with complete case analyses [43], however, some residual bias could remain due to the nature of some of the missing data. The associations between maternal characteristics and dispensing patterns in our cohort were weak. Some studies have found stronger associations between the use of at least one medicine during pregnancy and BMI [5], smoking [44], ethnicity [45], and household income [14], although observed relationships with parity [5, 18, 44] and maternal age [5, 44] vary. However, different settings and health systems from the New Zealand context make direct comparisons with international studies difficult.

The weak association with maternal characteristics in this study could be due to residual confounding or a true reflection that dispensing in pregnancy in New Zealand is not strongly dependent on these factors. Antenatal care is free, with midwives able to prescribe within their scope of practice, partially reducing the impact of recognised financial barriers associated with accessing primary care [46]. We were unable to adjust for factors such as education level, household income, alcohol use, marital status, and whether a pregnancy was planned, which have been shown to be associated with medicine use in pregnancy in other studies [14, 44, 45]. Other studies have found an association between a summary measure of chronic health conditions and being dispensed a prescription medicine during pregnancy [5, 45]. We were not able to explore this association because we did not have access to primary care data and we did not think the use of hospitalisation records would provide a reliable measure of chronic disease in women of child-bearing age. Moreover, we felt that using dispensings of medicines to generate a chronic condition variable to then analyse dispensing patterns would be circular.

The proportion of pregnancies ending in a delivery during which a non-supplement medicine was dispensed in New Zealand (64.6%) occurred in the middle of the range of international comparisons over a similar time period, and was roughly comparable to exposure in British Columbia, Canada (2002–2011: 62%) [5] and Denmark (1999–2009: 56.0%) [4]. Substantially higher exposure was found for women in the US Medicaid Program (2000–2007: 82.5%) [9] and in France (2004–2005: 93%) [13]. Women with pregnancies ending in a delivery in New Zealand were exposed to a similar mean number of different medicines during pregnancy (3.1) as those in British Columbia (2.7) [15], Denmark (2.6) [14] and Norway (3.3) [10], but much lower than French women (11.3) [13]. Medicines requiring a prescription may vary between countries, which could contribute to the variation in these estimates across studies.

Our most commonly dispensed non-supplement group during pregnancy was Antibacterials (26.0% during pregnancy; 11.0%–14.4% per trimester), which was also the most dispensed group during pregnancy in Western Australia (10.4%) [18] and a number of European (11.6%–27.0% during pregnancy [19, 21]; 9.4%–12.5% per trimester [8, 10]) and North American (26.1%–39.8%) [15–17] locations. Antibacterials were also highly dispensed during pregnancy in the Netherlands (20.6%) [23] and France (50.9%) [47]. Pregnancy exposure to Analgesics (including opioids and non-opioids) in New Zealand (16.7%; 6.7%–9.6% per trimester) was similar to that found in a study of eight Health Maintenance Organizations (14.2%) in the United States [17], although that study’s timeframe (1996–2000) was prior to the opioid epidemic [48]. Except for in France (72.0%) [47] analgesic exposure was much lower in most other regions (3.7% [18], 4.3% [23]; 1–1.5% per trimester [49]) than in New Zealand. Our use of Antinausea & Vertigo Agents (11.0%) was similar to that in Quebec (13.7%) [16] but direct comparisons were more difficult as some locations [8, 10, 15, 23] had lower antiemetic use but higher use of antihistamines, some of which (e.g. doxylamine) may be used for alleviating nausea in pregnancy.

## Conclusion

This high-level overview of dispensing patterns has demonstrated increasingly medicated pregnancies in New Zealand in recent years, which is consistent with international trends in medicine use both within [4–6] and outside of [50, 51] pregnancy. Like their non-pregnant counterparts, pregnant women deserve to have their health issues treated effectively, so while this increase in medicine use isn’t necessarily cause for concern, the lack of pregnancy safety information for most medicines does mean that this increase shouldn’t be ignored. This study further highlights the urgent need for researchers to better quantify potential fetal impacts of exposure to medications commonly used during pregnancy.

This study provides directions for future research. For example, Antibacterials were dispensed in more than a quarter of New Zealand pregnancies, and it would be informative to investigate the specific antibiotics dispensed as some (tetracyclines, aminoglycosides) are not recommended in pregnancy. The high prevalence of anti-emetic exposure in the first trimester (8%) could also warrant further investigation, considering recent questions about the fetal safety of ondansetron in early pregnancy [52–54].

## Supporting information

S1 File Assignment of maternal characteristics. (PDF)

S2 File Details of the multiple imputation model to estimate missing maternal characteristics. (PDF)

S1 Fig Pre-pregnancy and pregnancy time periods examined in this study. (PDF)

S1 Table Products in the Pharmaceutical Collection excluded from this study. (PDF)

S2 Table Products in the Pharmaceutical Collection categorised as supplements. (PDF)

S3 Table Number of pregnancies per cohort member. (PDF)

S4 Table Pregnancy outcomes by study year. (PDF)

S5 Table Proportion with ≥1 dispensing of a non-supplement medicine, for the whole cohort and for pregnancies ending in a delivery. (PDF)

S6 Table Comparison of the proportion of exposed pregnancies using the earliest vs latest LMP dates. (PDF)

S7 Table Comparison of the proportions with ≥1 non-supplement dispensing in pre-pregnancy using the whole cohort vs left-censoring for those with a previous pregnancy within 270 days. (PDF)

S8 Table Proportions with ≥1 dispensing of a non-supplement medication by maternal characteristics: complete case analyses vs analyses using imputed data, by trimester. (PDF)

S9 Table Proportions with ≥1 dispensing from Level 2 therapeutic groups; trends before and during pregnancy (with relative risks and 95% confidence intervals). (PDF)

S10 Table Proportions with ≥1 dispensing from Level 2 therapeutic groups; trends over study years (with relative risks and 95% confidence intervals). (PDF)

S11 Table Proportions with ≥1 dispensing from the Level 2 therapeutic groups before and during pregnancy. Table includes all therapeutic groups dispensed to at least one cohort member.
(PDF)

S12 Table Proportions with ≥1 dispensing from Level 2 therapeutic groups during Trimester 1, by grouped pregnancy outcome. (PDF)
