# Supplementary material for: Patterns of prescription medicine dispensing before and during pregnancy in New Zealand, 2005–2015
Source: PLoS One. 2020 Jun 2;15(6):e0234153. doi: 10.1371/journal.pone.0234153 (PMC7266349; doi:10.1371/journal.pone.0234153)
Supplement: S1 File — (PDF) [file pone.0234153.s001.pdf]

S1 Assignment of maternal characteristics

Maternal age

The maternal age at the last menstrual period was calculated by subtracting the mother's date of birth recorded in the National Health Index (NHI) Collection from her LMP date.

Prioritised ethnicity

In New Zealand ethnicity is self-identified, and a person may identify with more than one ethnic group. Our data extract from the national health databases held information on up to three different ethnicities per person. Commonly in health research, a single, prioritised ethnicity is allocated to an individual to ensure that an individual is only counted once.[1]

Each woman in our cohort was assigned a single prioritised Level 1 ethnicity according to Statistics NZ categories[1] (Table A1.1), according to the highest prioritised ethnicity ever recorded in our data extracts from the NHI Collection, the Maternity Collection (MAT), the National Minimum Dataset (NMDS - hospitalisations), the Mortality Collection (MORT), the Laboratory Claims Collection (LAB) and the Pharmaceutical Collection (PHARMS). This method was chosen to minimise undercounting of non-European ethnic groups, and to ensure consistency for all pregnancies to an individual woman. Residual Categories were counted as missing.

Table A1.1 Statistics NZ prioritisation order of Level 1 ethnic groups

Priority order	Level 1 ethnic group
1	Māori
2	Pacific
3	Asian
4	Middle Eastern/Latin American/African
5	Other ethnicity
6	European
9	Residual Categories†

† *Residual categories includes answers such as "don't know", "refused to answer", and "response outside scope"*

Deprivation (NZDep) quintile

The New Zealand Deprivation Index (NZDep) is a measure of small-area (neighbourhood) deprivation[2] generated from information gathered in the 5-yearly national Census. It is derived from a number of variables (e.g. household income, home ownership, employment status, internet access) and is updated following each Census. The population is divided into 10 deciles, with areas designated as decile 1 being the least deprived 10% of areas in New Zealand and decile 10 representing the most deprived 10%.

Cohort members were assigned their NZDep score recorded in MAT (recorded at delivery), and if that was not available, from their records in the other source collections. To reduce the number of deprivation groups for analysis the NZDep deciles were converted into quintiles. For women with an LMP in 2005, NZDep01 was used. NZDep06 was used for pregnancies with LMPs from 2006–2012, and NZDep13 for pregnancies with LMPs in 2013-2015.

Body Mass Index

BMI information was only available in MAT, and only for records originating from lead maternity carer (LMC) claims. BMI is a derived variable, calculated from weight and height information recorded at the first antenatal visit with the LMC. Implausible BMI values (the bottom and top 0.1%) were not retained. BMI values were categorised as underweight (<18.5), normal weight (18.5–<25), overweight (25–<29) and obese (≥30).

Smoking status

Smoking information was only available in MAT and the NMDS. MAT contains a number of variables related to smoking. At the time of registration with her lead maternity carer (LMC) and at two weeks post-partum the LMC records the woman's smoking status (yes/no), and also the number of cigarettes smoked daily if she is a smoker. If the woman delivered in hospital, her admission (NMDS) record should record if she was a smoker at the time of delivery using the ICD-10-AM Z72.0 (tobacco use, current) code (although recording is only robust from 2008/09); these smoking data are transferred from NMDS to MAT along with other delivery information. Unfortunately non-smoker status is not recorded in the NMDS. Smoking status for women with an NMDS record who did not appear in MAT was obtained directly from the NMDS from the end of pregnancy admission using the Z72.0 code.

Many pregnancies in MAT did not have complete smoking data, therefore a composite smoking variable was created to indicate whether the woman was a smoker at the start of pregnancy. A woman recorded as a smoker in early pregnancy or at 2 weeks postpartum, or having or cigarettes smoked variables was classified as a smoker, even if she was recorded as a non-smoker in one of the variables. We made the assumption that a non-smoker would be very unlikely to start smoking during pregnancy, so that a positive smoking status at delivery or post-partum was a good proxy for positive smoking status at pregnancy start. Women with a non-smoker status specifically recorded by the LMC in MAT and who had no positive smoking variables recorded in the NMDS was classified

as a non-smoker. Smoking status was left blank for women with no positive smoking variables and who were not specified as a non-smoker.

Parity

Women were identified as primiparous (‘primips’) or non-primiparous (‘non-primips’). Parity information was obtained from MAT and NMDS. MAT contains a parity variable but the values were not always accurate. MAT records (available back to 2005) were reviewed to find the earliest recorded delivery for each woman. NMDS records (available back to 1988) were searched for any deliveries to cohort members.

The earliest recorded pregnancy ending in a delivery for an individual woman was identified where possible. Women were categorised as a primip if their MAT record identified them as a primip and there were no identified deliveries prior to the pregnancy in question. Women with an identified prior delivery were termed a non-primip for all subsequent pregnancies irrespective of whether their MAT record had recorded them as a primip. Women without an identified prior delivery who were recorded as a non-primip in MAT were categorised as a non-primip for that pregnancy. The parity variable was left blank for pregnancies with no previously identified delivery and no information on parity recorded in MAT.

References

1. Ministry of Health. *HISO 10001:2017. Ethnicity data protocols.* Wellington: Ministry of Health;2017.
2. Atkinson J, Salmond C, Crampton P. *NZDep2013 Index of Deprivation* Wellington: Department of Public Health, University of Otago;2014.
