# Supplementary material for: Patterns of prescription medicine dispensing before and during pregnancy in New Zealand, 2005–2015
Source: PLoS One. 2020 Jun 2;15(6):e0234153. doi: 10.1371/journal.pone.0234153 (PMC7266349; doi:10.1371/journal.pone.0234153)
Supplement: S2 File — (PDF) [file pone.0234153.s002.pdf]

S5 Details of the multiple imputation model to estimate missing maternal characteristics

Multiple imputation was used to estimate sociodemographic information for cohort members with missing values – BMI (48.0%), smoking status (36.7%), parity (21.9%), ethnicity (0.2%) and deprivation (NZDep) quintile (0.1%). The data used to create the cohort and source the maternal characteristics were obtained from four different national databases. Data were missing for a combination of reasons, including the specific database from which a pregnancy was sourced, the year the pregnancy occurred, as well as individual decisions made by those responsible for collecting the data at the point of care. The nature of the missing data is most likely a combination of missing at random (MAR) and missing not at random (MNAR).

For example, BMI information was missing for all pregnancies not sourced from the maternity database, therefore much of the missing data is related to the data source and, by extension, the outcome of pregnancy. Within the maternity database, some records were also missing a BMI value. Maternity records are acquired from two sources – claims from lead maternity carers (LMCs – predominantly independent midwives) for antenatal services, and hospitalisation records for obstetric care. Only records from the LMC claims include BMI information. For women with an independent midwife, the likelihood of having a BMI recorded could vary by the midwife's personal views of recording pregnant women's weight in general, or by a reluctance to enquire about weight with heavier women, or a combination of factors. Additionally, BMI information was missing for almost all maternity records prior to 2007, with coverage improving to over 95% by 2013.

We undertook similar explorations of the missing data for the other maternal characteristics, and are satisfied that multiple imputation was a valid choice for estimating complete data sets prior to analyses, as the maternal characteristics and auxiliary variables included in the imputation model should allow reasonable estimations of the missing values to be generated.

The first step in building our imputation model involved the identification of auxiliary variables - variables which are associated with the missing values and improve the predictive ability of the model, but which are not included in the analytic models. A number of medicines and diagnoses in the dispensing (Table A4.1) and hospitalisation (Table A4.2) databases were assessed for association (either positively or negatively) with the maternal characteristics with incomplete data, especially smoking status and BMI. Cohort members with at least one coded diagnosis or one dispensing <1 year prior to conception, 1 to <2 years prior to conception, 2 to <5 years prior to conception or 5 to 10 years (diagnoses only) prior to conception were identified, and a binary yes/no variable was created for each of the diagnoses/medicines for each time period.

Because dispensing data were only robust from 2005 onward, some cohort members did not have 5 complete years of pre-pregnancy dispensing history. For each of the time periods considered for the auxiliary variables, any cohort member with at least 1 dispensing in a time period was assigned a 'yes' value for that time period. However, cohort members whose available dispensing history did not cover the whole time period of interest, and who did not have a dispensing in the portion of the period for which they did have information, were assigned a missing value (as we could not exclude that there had been a dispensing pre-2005). For example, a cohort member with an LMP in June 2005 was assigned 'missing' values for their auxiliary dispensing variables for 1 to <2 years and 2 to <5 years prior to pregnancy, because these periods finished prior to 2005. If there was no

dispensing between January and June 2005 they were assigned a missing value for the <1 year dispensing variable, as there could have been a dispensing in the last half of 2004. However, if they did have a dispensing between January and June 2005, they would have been classified as 'yes' for ≥1 dispensing in the year prior to pregnancy.

Table A4.1 Dispensings assessed for correlation with maternal characteristics

Treatment type	Medicines	PHARMS code			
		TG1	TG2	TG3	Chemical ID
Smoking cessation medications	Nicotine	22	34	1	3722
	Bupropion	22	34	1	3892
	Varenicline	22	34	1	3920
Type 2 diabetes medications	Alpha glucosidase inhibitors	1	13	11	
	Oral hypoglycaemics	1	13	12	
Respiratory medications	Inhaled Corticosteroids - Metered Dose Inhalers	28	7		
	Inhaled Corticosteroids	28	10		
	Inhaled Long-acting Beta-adrenoceptor Agonists	28	24		
	Beta-Adrenoceptor Agonists	28	30		
	Anticholinergic Agents	28	34		
	Leukotriene Receptor Antagonists	28	37		
	Other Bronchodilators	28	45		
	Respiratory Devices	28	53		
Lipid lowering medications	Lipid-Modifying Agents	7	32		

Table A4.2 Diagnoses assessed for correlation with sociodemographic variables

Diagnosis	ICD-10-AM code
Type 2 diabetes	E11
Polycystic ovary syndrome	E28.2
Obesity	E66
Cystic fibrosis	E84
Mental and behavioural disorders due to use of tobacco	F17
Obstructive sleep apnoea	G47.32
Chronic bronchitis	J41/J42
Chronic obstructive pulmonary disease	J43/J44
Asthma	J45/J46
Bronchiectasis	J47
Tobacco use, current	Z72.0[a]

[a] *The same ICD-10-AM code contributed to the generation of the smoking status variable, but the timeframes for the auxiliary variable (prior to pregnancy) and the maternal characteristic (at the end-of-pregnancy admission) were different*

The potential auxiliary variables were then assessed for association with the sociodemographic variables to be imputed. The most highly-associated variables (those with an absolute Z score of 4

or more (for which P<0.001)) were chosen for inclusion in the model as auxiliary variables. Auxiliary dispensing variables with missing data were also imputed in the model.

The final imputation model included the five sociodemographic variables to be imputed, all other predictor variables to be included in the regression analyses, the auxiliary variables and three outcome variables (Table A4.3). Three outcome variables were included as the imputed data were to be used for separate analyses of non-supplement dispensings in pregnancy (current study) as well as analyses of Category D and Category X medicine dispensings (a separate study, not included in this manuscript).

The variables to be imputed were a combination of binary, ordinal and categorical, therefore multiple imputation was performed using chained equations, which allows a separate conditional distribution to be specified for each type of imputed variable. The multiple imputation was undertaken with m=40, and a burn-in period of 10 iterations. Following the imputation phase, the convergence of the imputed values for each of the imputed sociodemographic variables was visually assessed using trace plots of the estimated parameters against iteration numbers for the burn-in period, which all appeared to converge to stationarity by 10 iterations. Analyses using the imputed datasets were then undertaken.

Table A4.3 Variables included in the imputation model

**Predictor variables**	**% Missing**	**Imputed**	**Distribution**
Year of LMP	-	-	-
Age group category	-	-	-
Ethnicity	0.2	yes	mlogit
Deprivation category	0.1	yes	ologit
Smoking status	36.7	yes	logit
Body mass index category	48.0	yes	ologit
Parity	21.9	yes	logit
**Auxiliary variables**	**% Missing**	**Imputed**	**Distribution**
Smoking cessation medication <1 year prior to pregnancy	3.1	yes	logit
Smoking cessation medication 1-<2 years prior to pregnancy	14.1	yes	logit
Smoking cessation medication 2-<5 years prior to pregnancy	47.2	yes	logit
Diabetes medication <1 year prior to pregnancy	3.1	yes	logit
Diabetes medication 1-<2 years prior to pregnancy	14.1	yes	logit
Diabetes medication 2-<5 years prior to pregnancy	47.2	yes	logit
Respiratory medication <1 year prior to pregnancy	2.9	yes	logit
Respiratory medication1-<2 years prior to pregnancy	13.6	yes	logit
Respiratory medication 2-<5 years prior to pregnancy	44.4	yes	logit
Lipid lowering medication <1 year prior to pregnancy	3.1	yes	logit
Obesity coded <1 year prior to pregnancy	-	-	-
Obesity coded 1-<2 years prior to pregnancy	-	-	-
Obesity coded 2-<5 years prior to pregnancy	-	-	-
Obesity coded 5-10 years prior to pregnancy	-	-	-
Tobacco use coded <1 year prior to pregnancy	-	-	-
Tobacco use coded 1-<2 years prior to pregnancy	-	-	-
Tobacco use coded 2-<5 years prior to pregnancy	-	-	-
Tobacco use coded 5-10 years prior to pregnancy	-	-	-
Asthma coded <1 year prior to pregnancy	-	-	-
Asthma coded 1-<2 years prior to pregnancy	-	-	-
Asthma coded 2-<5 years prior to pregnancy	-	-	-
Asthma coded 5-10 years prior to pregnancy	-	-	-
Diabetes coded <12 months prior to pregnancy	-	-	-
Diabetes coded 5-10 years prior to pregnancy	-	-	-
Polycystic ovary syndrome coded <1 year prior to pregnancy	-	-	-
Polycystic ovary syndrome coded 1-<2 years prior to pregnancy	-	-	-
Polycystic ovary syndrome coded 5-10 years prior to pregnancy	-	-	-
Obstructive sleep apnoea 5-10 years prior to pregnancy	-	-	-
**Outcome variables**[a]	**% Missing**	**Imputed**	**Distribution**
Number of different medicines dispensed during pregnancy	-	-	-
Number of different Category D medicines dispensed during pregnancy	-	-	-
Number of different Category X medicines dispensed during pregnancy	-	-	-

[a] *Dispensing of Category D/X medicines will be reported in a separate manuscript*
