# Supplementary material for: Patterns of prescription medicine dispensing before and during pregnancy in New Zealand, 2005–2015
Source: PLoS One. 2020 Jun 2;15(6):e0234153. doi: 10.1371/journal.pone.0234153 (PMC7266349; doi:10.1371/journal.pone.0234153)

S4 Pre-pregnancy and pregnancy time periods examined in this study

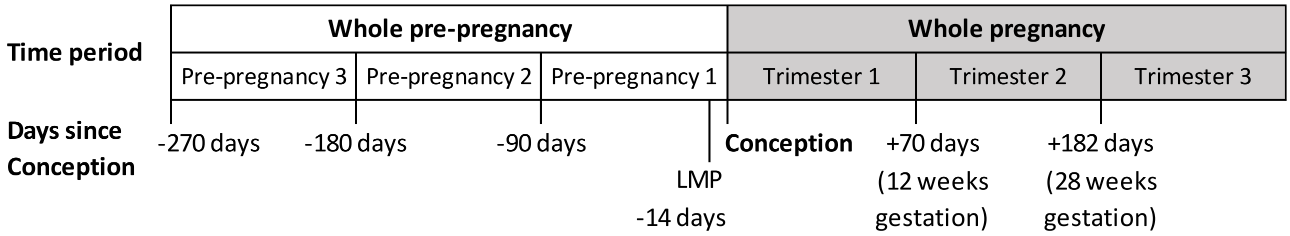

Supplement: S1 Fig — (PDF) [file pone.0234153.s003.pdf]
