# Supplementary material for: Patterns of prescription medicine dispensing before and during pregnancy in New Zealand, 2005–2015
Source: PLoS One. 2020 Jun 2;15(6):e0234153. doi: 10.1371/journal.pone.0234153 (PMC7266349; doi:10.1371/journal.pone.0234153)
Supplement: S1 Table — (PDF) [file pone.0234153.s004.pdf]

S2 Products in the Pharmaceutical Collection excluded from this study

Where only Therapeutic Group Level 1 or Levels 1 and 2 are shown, all items contained within the group were excluded. Where Therapeutic Group Level 3 or chemical id are shown, only those specific Level 3 groups or chemicals were excluded. All substances within the Level 1 group *Extemporaneously Compounded Preparations and Galenicals* were excluded, except for the chemicals listed, which were <u>included</u> in the analyses.

<table>
<tr><th>Therapeutic Group Level 1</th><th>Therapeutic Group Level 2</th><th>Therapeutic Group Level 3</th><th>Chemical Name</th></tr>
<tr><td>Unknown</td><td>Unknown</td><td colspan="2"></td></tr>
<tr><td>Alimentary Tract and Metabolism</td><td>Diabetes Management[a]</td><td colspan="2"></td></tr>
<tr><td rowspan="2">Blood and Blood Forming Organs</td><td>Antithrombotic Agents</td><td>Antiplatelet Agents</td><td>Coag U Chek</td></tr>
<tr><td>Fluids and Electrolytes</td><td colspan="2"></td></tr>
<tr><td rowspan="15">Dermatologicals</td><td>Antipruritic Preparations</td><td colspan="2"></td></tr>
<tr><td>Disinfecting and Cleansing Agents</td><td colspan="2"></td></tr>
<tr><td>Dusting Powders</td><td colspan="2"></td></tr>
<tr><td>Barrier Creams and Emollients</td><td colspan="2"></td></tr>
<tr><td>Minor Skin Infections</td><td colspan="2"></td></tr>
<tr><td rowspan="7">Psoriasis and Eczema Preparations</td><td rowspan="7">Psoriasis and Eczema Preparations</td><td>Coal tar with allantoin, menthol, phenol and sulphur</td></tr>
<tr><td>Pine tar with trolamine laurilsulfate and fluorescein</td></tr>
<tr><td>Tar with cade oil</td></tr>
<tr><td>Coal tar</td></tr>
<tr><td>Potassium permanganate</td></tr>
<tr><td>Sulphur</td></tr>
<tr><td>Evening Primrose Oil</td></tr>
<tr><td colspan="3">Sunscreens</td></tr>
<tr><td rowspan="2">Other Skin Preparations</td><td>Antiperspirants</td><td></td></tr>
<tr><td>Wound Management Products</td><td></td></tr>
<tr><td rowspan="3">Genito-Urinary System</td><td>Contraceptives - Non-hormonal</td><td colspan="2"></td></tr>
<tr><td>Pregnancy Tests - hCG Urine</td><td colspan="2"></td></tr>
<tr><td>Detection of Substances in Urine</td><td colspan="2"></td></tr>
</table>

Therapeutic Group Level 1	Therapeutic Group Level 2	Therapeutic Group Level 3	Chemical Name
Respiratory System and Allergies	Antiallergy Preparations		
	Respiratory Devices		
Various	Various		
Extemporaneously Compounded Preparations and Galenicals	**All excluded, except for chemical names listed at the right (which were included)**		Ascorbic acid
			Codeine phosphate
			Methadone hydrochloride
			Paracetamol
			Phenobarbitone sodium
			Magnesium hydroxide
			Benzyl benzoate
			Chloral hydrate
			Chloroform
			Chloroform water concentrate
			Dithranol
			Podophyllum resin
Special Foods			
National Immunisation Schedule	Vaccinations		

[a] *Diabetes Management only contains non-medicinal products used for diabetes management, such as syringes, insulin pumps and related consumables, and blood glucose test strips. Insulin, oral hypoglycaemics and other pharmaceuticals used to treat diabetes are in a separate Level 2 therapeutic group (Diabetes), which was included in the study.*
