# Supplementary material for: Patterns of prescription medicine dispensing before and during pregnancy in New Zealand, 2005–2015
Source: PLoS One. 2020 Jun 2;15(6):e0234153. doi: 10.1371/journal.pone.0234153 (PMC7266349; doi:10.1371/journal.pone.0234153)
Supplement: S2 Table — (PDF) [file pone.0234153.s005.pdf]

S3 Products in the Pharmaceutical Collection categorised as supplements

Therapeutic Group Level 1	Therapeutic Group Level 2	Therapeutic Group Level 3	Chemical Name
Alimentary Tract and Metabolism	Vitamins	Vitamin A	
		Vitamin B	
		Vitamin C	
		Vitamin D	
		Vitamin E	
		Multivitamin Preparations	
	Minerals	Calcium	
		Magnesium	
		Calcium	
		Fluoride	
		Iodine	
		Iron	
		Magnesium	
		Zinc	
Blood and Blood Forming Organs	Antianaemics[a]	Iron Therapy	Coenzyme Q (Ubiquinone)
			Ferrous gluconate
		Megaloblastic	Cyanocobalamin (B12)
			Folic acid
Extemporaneously Compounded Preparations and Galenicals	Extemporaneously Compounded Preparations and Galenicals	Extemporaneously Compounded Preparations and Galenicals	Ascorbic acid

[a] *Antianaemics which were not categorised as supplements include erythropoietin alfa/beta, filgrastim, arginine hydrochloride, molgramostin and sodium phenylbutyrate*
