# Supplementary material for: Patterns of prescription medicine dispensing before and during pregnancy in New Zealand, 2005–2015
Source: PLoS One. 2020 Jun 2;15(6):e0234153. doi: 10.1371/journal.pone.0234153 (PMC7266349; doi:10.1371/journal.pone.0234153)
Supplement: S3 Table — (PDF) [file pone.0234153.s006.pdf]

S6 Number of pregnancies per cohort member

Number of pregnancies in cohort	Number of women	Proportion (%) of women
1	224,030	47.8
2	139,441	29.8
3	66,047	14.1
4	26,142	5.6
5	9,032	1.9
6	2,753	0.6
7	773	0.2
8	203	0.0
9	45	0.0
10	12	0.0
11	1	0.0
12	0	0.0
13	0	0.0
14	1	0.0
