# Supplementary material for: Patterns of prescription medicine dispensing before and during pregnancy in New Zealand, 2005–2015
Source: PLoS One. 2020 Jun 2;15(6):e0234153. doi: 10.1371/journal.pone.0234153 (PMC7266349; doi:10.1371/journal.pone.0234153)
Supplement: S4 Table — (PDF) [file pone.0234153.s007.pdf]

S7 Pregnancy outcomes by study year

Pregnancy outcome	Proportion (%) of yearly pregnancies										
	2005	2006	2007	2008	2009	2010	2011	2012	2013	2014	2015
Deliveries (live and stillborn infants)	65.2	65.4	65.2	65.2	67.6	67.7	67.3	67.8	68.0	68.9	65.6
Miscarriages/other early losses	6.1	5.7	5.6	5.8	5.7	5.7	5.8	5.9	5.9	5.7	5.1
Terminations	8.7	8.9	8.7	8.7	9.5	10.6	10.1	9.4	8.9	8.4	8.0
Undetermined	20.1	20.0	20.4	20.4	17.1	16.0	16.8	16.9	17.2	17.0	21.3
