# Supplementary material for: Patterns of prescription medicine dispensing before and during pregnancy in New Zealand, 2005–2015
Source: PLoS One. 2020 Jun 2;15(6):e0234153. doi: 10.1371/journal.pone.0234153 (PMC7266349; doi:10.1371/journal.pone.0234153)
Supplement: S5 Table — (PDF) [file pone.0234153.s008.pdf]

S8 Proportion (%) with ≥1 dispensing of a non-supplement medicine, for the whole cohort and for pregnancies ending in a delivery

A) All cohort pregnancies

LMP year	Pre-preg 3[a]	Pre-preg 2[b]	Pre-preg 1[c]	Trimester 1	Trimester 2[d]	Trimester 3[d]
2005	26.2	27.6	25.6	22.6	23.7	24.9
2006	29.2	28.6	26.7	24.8	25.7	24.2
2007	30.9	31.1	29.9	29.2	30.6	28.5
2008	35.5	35.6	33.7	32.5	35.3	34.7
2009	38.1	38.2	36.1	35.1	38.6	38.2
2010	40.0	39.6	37.2	37.2	40.8	41.1
2011	41.4	41.0	39.0	39.3	43.9	43.5
2012	42.5	41.9	39.8	40.4	45.1	44.5
2013	42.4	41.3	39.2	41.0	45.4	45.0
2014	41.8	41.4	39.6	41.8	46.4	46.5
2015	43.6	43.2	38.7	42.0	48.4	47.3
Total	37.5	37.3	35.3	35.2	38.6	38.1

B) Deliveries only (includes deliveries of live and stillborn infants)

LMP year	Pre-preg 3[a]	Pre-preg 2[b]	Pre-preg 1[c]	Trimester 1	Trimester 2[d]	Trimester 3[d]
2005	25.8	26.3	23.9	21.3	25.2	25.1
2006	28.4	27.3	25.1	23.4	27.3	24.4
2007	29.8	29.8	28.2	28.0	32.9	28.8
2008	34.6	34.3	32.2	31.5	37.8	35.0
2009	37.6	37.3	34.7	34.4	41.1	38.6
2010	39.1	38.3	35.6	36.4	43.1	41.5
2011	40.6	39.7	37.7	38.7	45.8	44.1
2012	41.7	40.8	38.1	39.8	47.0	45.1
2013	41.6	40.2	37.8	40.8	47.1	45.7
2014	40.7	40.1	38.0	41.8	48.2	47.0
2015	42.5	41.6	37.3	42.7	50.4	49.0
Total	36.8	36.1	33.8	34.6	40.7	38.5

[a] *181-270 days prior to conception*

[b] *91-180 days prior to conception*

[c] *1-90 days prior to conception*

[d] *Denominator only includes pregnancies that persisted to the start of the trimester*
