# Supplementary material for: Patterns of prescription medicine dispensing before and during pregnancy in New Zealand, 2005–2015
Source: PLoS One. 2020 Jun 2;15(6):e0234153. doi: 10.1371/journal.pone.0234153 (PMC7266349; doi:10.1371/journal.pone.0234153)
Supplement: S6 Table — (PDF) [file pone.0234153.s009.pdf]

S9 Comparison of the proportion of exposed pregnancies using the earliest vs latest LMP dates

The proportion of pregnancies with at least one dispensing of a non-supplement prescription medicine during the different pre-pregnancy and pregnancy periods using the earliest LMP dates

Year of LMP†	**Pre-pregnancy**												**Pregnancy**											
	**Pre-pregnancy 3**			**Pre-pregnancy 2**			**Pre-pregnancy 1**			**Whole Pre-pregnancy**			**Trimester 1**			**Trimester 2**			**Trimester 3**			**Whole Pregnancy**		
	%	RR‡	95% CI	%	RR‡	95% CI	%	RR‡	95% CI	%	RR‡	95% CI	%	RR‡	95% CI	%§	RR‡	95% CI	%§	RR‡	95% CI	%	RR‡	95% CI
2005	26.2	*reference*		27.6	*reference*		25.6	*reference*		47.0	*reference*		22.6	*reference*		23.7	*reference*		24.9	*reference*		38.5	*reference*	
2006	29.2	1.11	[1.09-1.14]	28.6	1.04	[1.02-1.06]	26.7	1.04	[1.02-1.06]	49.6	1.05	[1.04-1.07]	24.8	1.10	[1.07-1.12]	25.7	1.08	[1.05-1.11]	24.2	0.97	[0.95-1.00]	40.7	1.06	[1.04-1.08]
2007	30.9	1.18	[1.15-1.21]	31.1	1.13	[1.10-1.15]	29.9	1.17	[1.14-1.19]	53.7	1.14	[1.13-1.16]	29.2	1.29	[1.26-1.32]	30.6	1.29	[1.26-1.33]	28.5	1.15	[1.12-1.18]	48.0	1.25	[1.23-1.27]
2008	35.5	1.36	[1.33-1.38]	35.6	1.29	[1.27-1.32]	33.7	1.32	[1.29-1.35]	59.9	1.27	[1.26-1.29]	32.5	1.44	[1.40-1.47]	35.3	1.49	[1.45-1.53]	34.7	1.40	[1.36-1.43]	54.1	1.41	[1.38-1.43]
2009	38.1	1.45	[1.42-1.49]	38.2	1.39	[1.36-1.42]	36.1	1.41	[1.38-1.44]	63.5	1.35	[1.33-1.37]	35.1	1.55	[1.51-1.59]	38.6	1.63	[1.59-1.67]	38.2	1.54	[1.49-1.58]	58.6	1.52	[1.50-1.55]
2010	40.0	1.53	[1.50-1.56]	39.6	1.44	[1.41-1.47]	37.2	1.45	[1.42-1.48]	65.5	1.39	[1.37-1.41]	37.2	1.64	[1.60-1.68]	40.8	1.72	[1.68-1.77]	41.1	1.65	[1.61-1.70]	61.7	1.60	[1.58-1.63]
2011	41.4	1.58	[1.54-1.61]	41.0	1.48	[1.45-1.52]	39.0	1.52	[1.49-1.56]	67.1	1.43	[1.41-1.45]	39.3	1.74	[1.70-1.78]	43.9	1.85	[1.80-1.90]	43.5	1.75	[1.70-1.80]	64.3	1.67	[1.65-1.70]
2012	42.5	1.62	[1.59-1.66]	41.9	1.52	[1.49-1.55]	39.8	1.55	[1.52-1.59]	68.0	1.45	[1.43-1.47]	40.4	1.78	[1.74-1.83]	45.1	1.90	[1.85-1.95]	44.5	1.79	[1.74-1.84]	65.8	1.71	[1.69-1.74]
2013	42.4	1.62	[1.58-1.65]	41.3	1.50	[1.47-1.53]	39.2	1.53	[1.50-1.57]	67.9	1.44	[1.43-1.46]	41.0	1.81	[1.77-1.85]	45.4	1.91	[1.86-1.96]	45.0	1.81	[1.76-1.86]	66.4	1.73	[1.70-1.75]
2014	41.8	1.59	[1.56-1.63]	41.4	1.50	[1.47-1.53]	39.6	1.55	[1.51-1.58]	67.6	1.44	[1.42-1.46]	41.8	1.85	[1.80-1.89]	46.4	1.96	[1.91-2.01]	46.5	1.87	[1.82-1.92]	67.5	1.75	[1.73-1.78]
2015	43.6	1.66	[1.62-1.71]	43.2	1.56	[1.53-1.61]	38.7	1.51	[1.47-1.55]	68.8	1.46	[1.44-1.49]	42.0	1.85	[1.80-1.90]	48.4	2.04	[1.98-2.10]	47.3	1.90	[1.84-1.96]	67.2	1.75	[1.72-1.78]
All years	37.5			37.3			35.3			61.9			35.2			38.6			38.1			57.8		

† Using the earliest LMP date for pregnancies with a range of LMP dates

‡ Adjusted for clustering by mother

§ Proportion of pregnancies that persisted to the start of the trimester

The proportion of pregnancies with at least one dispensing of a non-supplement prescription medicine during the different pre-pregnancy and pregnancy periods using the latest LMP dates.

Year of LMP†	Pre-pregnancy												Pregnancy											
	Pre-pregnancy 3			Pre-pregnancy 2			Pre-pregnancy 1			Whole Pre-pregnancy			Trimester 1			Trimester 2			Trimester 3			Whole Pregnancy		
	%	RR‡	95% CI	%	RR‡	95% CI	%	RR‡	95% CI	%	RR‡	95% CI	%	RR‡	95% CI	%§	RR‡	95% CI	%§	RR‡	95% CI	%	RR‡	95% CI
2005	26.3	*reference*		27.6	*reference*		25.6	*reference*		47.0	*reference*		21.8	*reference*		25.2	*reference*		24.8	*reference*		36.5	*reference*	
2006	29.1	1.11	[1.08 - 1.13]	28.4	1.03	[1.01 - 1.05]	26.7	1.04	[1.02 - 1.07]	49.6	1.05	[1.04 - 1.07]	23.7	1.09	[1.06 - 1.11]	27.2	1.08	[1.05 - 1.11]	24.2	0.97	[0.95 - 1.00]	38.4	1.05	[1.03 - 1.07]
2007	30.9	1.17	[1.15 - 1.20]	30.9	1.12	[1.10 - 1.15]	30.1	1.18	[1.15 - 1.20]	53.6	1.14	[1.13 - 1.16]	27.8	1.28	[1.25 - 1.31]	32.8	1.30	[1.27 - 1.34]	28.5	1.15	[1.12 - 1.18]	45.3	1.24	[1.22 - 1.26]
2008	35.6	1.35	[1.32 - 1.38]	35.4	1.28	[1.26 - 1.31]	33.9	1.33	[1.30 - 1.35]	60.0	1.28	[1.26 - 1.29]	31.4	1.44	[1.40 - 1.47]	37.6	1.49	[1.45 - 1.53]	34.6	1.39	[1.36 - 1.43]	51.3	1.41	[1.38 - 1.43]
2009	38.2	1.45	[1.42 - 1.48]	38.0	1.38	[1.35 - 1.41]	36.1	1.41	[1.38 - 1.44]	63.5	1.35	[1.33 - 1.37]	34.2	1.57	[1.53 - 1.61]	40.9	1.63	[1.58 - 1.67]	38.1	1.54	[1.50 - 1.58]	55.8	1.53	[1.51 - 1.56]
2010	40.1	1.52	[1.49 - 1.56]	39.4	1.43	[1.40 - 1.46]	37.4	1.46	[1.43 - 1.49]	65.5	1.39	[1.38 - 1.41]	36.4	1.67	[1.63 - 1.71]	42.9	1.71	[1.66 - 1.75]	41.1	1.66	[1.61 - 1.70]	58.6	1.61	[1.58 - 1.63]
2011	41.5	1.58	[1.54 - 1.61]	40.8	1.48	[1.45 - 1.51]	39.4	1.54	[1.51 - 1.58]	67.2	1.43	[1.41 - 1.45]	39.0	1.79	[1.75 - 1.83]	45.6	1.81	[1.77 - 1.86]	43.4	1.75	[1.70 - 1.80]	61.4	1.68	[1.66 - 1.71]
2012	42.4	1.61	[1.58 - 1.65]	41.7	1.51	[1.48 - 1.54]	40.0	1.57	[1.53 - 1.60]	68.1	1.45	[1.43 - 1.47]	40.2	1.84	[1.80 - 1.89]	46.9	1.86	[1.81 - 1.91]	44.4	1.79	[1.74 - 1.84]	63.1	1.73	[1.70 - 1.76]
2013	42.4	1.61	[1.58 - 1.64]	41.2	1.49	[1.46 - 1.53]	39.4	1.54	[1.51 - 1.58]	68.0	1.45	[1.43 - 1.47]	41.0	1.88	[1.84 - 1.93]	46.9	1.86	[1.82 - 1.91]	45.0	1.81	[1.77 - 1.86]	63.9	1.75	[1.72 - 1.78]
2014	41.7	1.59	[1.55 - 1.62]	41.2	1.50	[1.46 - 1.53]	39.8	1.56	[1.52 - 1.59]	67.7	1.44	[1.42 - 1.46]	41.7	1.91	[1.87 - 1.96]	48.0	1.91	[1.86 - 1.96]	46.4	1.87	[1.82 - 1.92]	64.9	1.78	[1.75 - 1.81]
2015	43.8	1.67	[1.62 - 1.71]	42.9	1.56	[1.52 - 1.60]	38.9	1.52	[1.48 - 1.57]	68.9	1.47	[1.44 - 1.49]	42.1	1.93	[1.88 - 1.99]	50.4	2.00	[1.94 - 2.06]	47.2	1.90	[1.84 - 1.96]	65.1	1.78	[1.75 - 1.82]
All years	37.6			37.1			35.4			62.0			34.5			40.5			38.0			55.1		

† Using the latest LMP date for pregnancies with a range of LMP dates

‡ Adjusted for clustering by mother

§ Proportion of pregnancies that persisted to the start of the trimester
