# Supplementary material for: Patterns of prescription medicine dispensing before and during pregnancy in New Zealand, 2005–2015
Source: PLoS One. 2020 Jun 2;15(6):e0234153. doi: 10.1371/journal.pone.0234153 (PMC7266349; doi:10.1371/journal.pone.0234153)
Supplement: S7 Table — (PDF) [file pone.0234153.s010.pdf]

S10 Comparison of the proportions with ≥1 non-supplement dispensing in pre-pregnancy using the whole cohort vs left-censoring the cohort for those with a previous pregnancy within 270 days

Year of LMP	Pre-pregnancy 3[a]			Pre-pregnancy 2[b]			Pre-pregnancy 1[c]			Whole Pre-pregnancy[d]		
	Whole cohort %	Left-censored cohort[e] %	Difference[f] %	Whole cohort %	Left-censored cohort[e] %	Difference[f] %	Whole cohort %	Left-censored cohort[e] %	Difference[f] %	Whole cohort %	Left-censored cohort[e] %	Difference[f] %
2005	26.2	26.2	0.0	27.6	27.5	0.1	25.6	25.6	0.0	47.0	47.0	0.0
2006	29.2	29.1	0.0	28.6	28.3	0.3	26.7	26.2	0.5	49.6	49.1	0.5
2007	30.9	30.4	0.5	31.1	30.1	1.0	29.9	28.9	1.0	53.7	52.1	1.6
2008	35.5	35.0	0.6	35.6	34.5	1.2	33.7	32.6	1.1	59.9	58.2	1.8
2009	38.1	37.0	1.1	38.2	36.5	1.7	36.1	34.8	1.2	63.5	61.3	2.3
2010	40.0	38.8	1.2	39.6	38.1	1.6	37.2	36.1	1.0	65.5	63.4	2.1
2011	41.4	39.9	1.5	41.0	39.1	1.8	39.0	37.9	1.1	67.1	64.8	2.3
2012	42.5	40.8	1.7	41.9	39.9	1.9	39.8	38.4	1.4	68.0	65.6	2.5
2013	42.4	40.5	1.8	41.3	39.3	2.0	39.2	37.8	1.4	67.9	65.4	2.5
2014	41.8	40.1	1.7	41.4	39.5	1.8	39.6	38.4	1.2	67.6	65.3	2.4
2015	43.6	42.0	1.6	43.2	41.3	1.8	38.7	37.3	1.4	68.8	66.6	2.2
All years	37.5	36.3	1.2	37.3	35.7	1.6	35.3	34.1	1.2	61.9	59.9	2.0

a Pre-pregnancy 3 covers the period 181–270 days prior to conception

b Pre-pregnancy 2 covers the period 91–180 days prior to conception

c Pre-pregnancy 1 covers the period 1–90 days prior to conception

de Whole Pre-pregnancy covers the period 1–270 days prior to conception

e Pregnancy excluded if the woman had a previous pregnancy within 270 days (n=126,713; 14.5%)

f Difference between proportion exposed in whole cohort vs the proportion exposed in the left-censored cohort
