# Supplementary material for: Patterns of prescription medicine dispensing before and during pregnancy in New Zealand, 2005–2015
Source: PLoS One. 2020 Jun 2;15(6):e0234153. doi: 10.1371/journal.pone.0234153 (PMC7266349; doi:10.1371/journal.pone.0234153)
Supplement: S8 Table — (PDF) [file pone.0234153.s011.pdf]

## S11 Proportions with ≥1 dispensing of a non-supplement medication by maternal characteristics: complete case analyses vs analyses using imputed data, by trimester

| Maternal characteristic | ≥1 dispensing of a non-supplement | | | | | |
|---|---|---|---|---|---|---|
| | Trimester 1 | | | | | |
| | Complete case analysis (n=447,460) | | | Imputed datasets (n=874,884) | | |
| | % | aRR[a] | [95% CI] | %[b] | aRR[a] | [95% CI] |
| **Age group (years)** | | | | | | |
| 15-19 | 36.5 | 1.05 | [1.03-1.07] | 36.5 | 1.06 | [1.05-1.07] |
| 20-29 | 36.0 | 1.00 | reference | 36.0 | 1.00 | reference |
| 30-39 | 33.8 | 0.94 | [0.93-0.95] | 33.8 | 0.95 | [0.95-0.96] |
| 40-49 | 37.2 | 1.00 | [0.98-1.03] | 37.2 | 1.03 | [1.01-1.05] |
| **Ethnicity (prioritised)[c]** | | | | | | |
| European | 35.2 | 1.00 | reference | 35.2 | 1.00 | reference |
| Māori | 36.0 | 0.94 | [0.93-0.95] | 36.0 | 0.92 | [0.92-0.93] |
| Pacific | 37.2 | 0.99 | [0.97-1.00] | 37.2 | 0.95 | [0.94-0.96] |
| Asian | 31.9 | 0.93 | [0.92-0.94] | 31.9 | 0.91 | [0.90-0.92] |
| MELAA[d] | 35.1 | 1.06 | [1.04-1.08] | 35.1 | 1.04 | [1.02-1.05] |
| Other | 31.9 | 0.93 | [0.77-1.13] | 31.9 | 0.91 | [0.79-1.05] |
| **NZDep quintile** | | | | | | |
| 1 (least deprived) | 32.5 | 1.00 | reference | 32.5 | 1.00 | reference |
| 2 | 33.5 | 1.01 | [1.00-1.03] | 33.5 | 1.02 | [1.01-1.03] |
| 3 | 34.4 | 1.04 | [1.02-1.05] | 34.4 | 1.04 | [1.03-1.05] |
| 4 | 36.1 | 1.07 | [1.06-1.09] | 36.1 | 1.08 | [1.07-1.09] |
| 5 (most deprived) | 37.2 | 1.08 | [1.06-1.09] | 37.2 | 1.09 | [1.08-1.10] |
| **BMI category** | | | | | | |
| Underweight (<18) | 33.9 | 1.01 | [0.99-1.04] | 31.2 | 0.98 | [0.95-1.00] |
| Healthy weight (18 - <25) | 33.5 | 1.00 | reference | 32.3 | 1.00 | reference |
| Overweight (25 - <30) | 37.5 | 1.10 | [1.09-1.11] | 36.2 | 1.10 | [1.09-1.11] |
| Obese (≥30) | 41.4 | 1.19 | [1.18-1.20] | 40.1 | 1.19 | [1.18-1.20] |
| **Smoking status** | | | | | | |
| Non-smoker | 35.2 | 1.00 | reference | 34.4 | 1.00 | reference |
| Smoker | 38.2 | 1.05 | [1.04-1.06] | 37.5 | 1.09 | [1.08-1.09] |
| **Parity** | | | | | | |
| Primiparous | 33.0 | 1.00 | reference | 33.4 | 1.00 | reference |
| Non-primiparous | 36.3 | 1.06 | [1.05-1.07] | 36.1 | 1.07 | [1.06-1.08] |

[a] *Adjusted for all other factors in the table, year of earliest LMP, and clustering by mother*

[b] *Proportions include imputed data (m=40)*

[c] *Ethnicity prioritised according to Statistics New Zealand Level 1 ethnic groups*

[d] *Middle Eastern/Latin American/African*

| Maternal characteristic | ≥1 dispensing of a non-supplement | | | | | |
|---|---|---|---|---|---|---|
| | Trimester 2 | | | | | |
| | Complete case analysis (n=447,443) | | | Imputed datasets (n=724,265) | | |
| | %[a] | aRR[b] | [95% CI] | %[ac] | aRR[a] | [95% CI] |
| **Age group (years)** | | | | | | |
| 15-19 | 40.7 | 1.12 | [1.11-1.14] | 40.7 | 1.07 | [1.06-1.08] |
| 20-29 | 39.2 | 1.00 | reference | 39.2 | 1.00 | reference |
| 30-39 | 37.4 | 0.98 | [0.97-0.98] | 37.4 | 0.98 | [0.97-0.99] |
| 40-49 | 39.7 | 1.05 | [1.03-1.08] | 39.7 | 1.01 | [0.99-1.02] |
| **Ethnicity (prioritised)[d]** | | | | | | |
| European | 37.8 | 1.00 | reference | 37.8 | 1.00 | reference |
| Māori | 40.9 | 0.96 | [0.95-0.97] | 40.9 | 1.00 | [0.99-1.01] |
| Pacific | 42.1 | 0.95 | [0.94-0.96] | 42.1 | 1.00 | [0.99-1.01] |
| Asian | 33.7 | 0.86 | [0.85-0.87] | 33.7 | 0.89 | [0.88-0.90] |
| MELAA[e] | 37.3 | 1.03 | [1.01-1.04] | 37.3 | 1.03 | [1.01-1.04] |
| Other | 30.8 | 0.89 | [0.75-1.06] | 30.8 | 0.82 | [0.70-0.95] |
| **NZDep quintile** | | | | | | |
| 1 (least deprived) | 35.2 | 1.00 | reference | 35.2 | 1.00 | reference |
| 2 | 36.4 | 1.02 | [1.00-1.03] | 36.4 | 1.02 | [1.01-1.03] |
| 3 | 37.5 | 1.04 | [1.02-1.05] | 37.5 | 1.04 | [1.03-1.06] |
| 4 | 38.9 | 1.06 | [1.04-1.07] | 38.9 | 1.07 | [1.06-1.08] |
| 5 (most deprived) | 42.0 | 1.08 | [1.07-1.09] | 42.0 | 1.11 | [1.10-1.13] |
| **BMI category** | | | | | | |
| Underweight (<18) | 37.6 | 0.99 | [0.97-1.01] | 33.2 | 0.97 | [0.95-0.99] |
| Healthy weight (18 - <25) | 38.7 | 1.00 | reference | 34.9 | 1.00 | reference |
| Overweight (25 - <30) | 43.9 | 1.10 | [1.10-1.11] | 39.7 | 1.10 | [1.09-1.11] |
| Obese (≥30) | 49.7 | 1.22 | [1.21-1.23] | 45.0 | 1.22 | [1.21-1.23] |
| **Smoking status** | | | | | | |
| Non-smoker | 40.7 | 1.00 | reference | 38.1 | 1.00 | reference |
| Smoker | 42.2 | 1.10 | [1.09-1.11] | 40.0 | 1.02 | [1.01-1.03] |
| **Parity** | | | | | | |
| Primiparous | 37.6 | 1.00 | reference | 36.8 | 1.00 | reference |
| Non-primiparous | 40.5 | 1.09 | [1.08-1.10] | 39.6 | 1.05 | [1.04-1.06] |

[a] *Denominator includes only those pregnancies that persisted to the start of the trimester*

[b] *Adjusted for all other factors in the table, year of earliest LMP, and clustering by mother*

[c] *Proportions include imputed data (m=40)*

[d] *Ethnicity prioritised according to Statistics New Zealand Level 1 ethnic groups*

[e] *Middle Eastern/Latin American/African*

| Maternal characteristic | ≥1 dispensing of a non-supplement | | | | | |
|---|---|---|---|---|---|---|
| | Trimester 3 | | | | | |
| | Complete case analysis (n=443,892) | | | Imputed datasets (n=590,660) | | |
| | %[a] | aRR[b] | [95% CI] | %[ac] | aRR[a] | [95% CI] |
| **Age group (years)** | | | | | | |
| 15-19 | 39.8 | 1.10 | [1.08-1.11] | 39.8 | 1.12 | [1.10-1.13] |
| 20-29 | 37.5 | 1.00 | reference | 37.5 | 1.00 | reference |
| 30-39 | 37.9 | 1.03 | [1.02-1.04] | 37.9 | 1.03 | [1.02-1.04] |
| 40-49 | 42.9 | 1.12 | [1.09-1.14] | 42.9 | 1.12 | [1.10-1.14] |
| **Ethnicity (prioritised)[d]** | | | | | | |
| European | 38.0 | 1.00 | reference | 38.0 | 1.00 | reference |
| Māori | 39.8 | 0.93 | [0.92-0.94] | 39.8 | 0.95 | [0.94-0.96] |
| Pacific | 39.6 | 0.90 | [0.89-0.91] | 39.6 | 0.93 | [0.92-0.94] |
| Asian | 33.4 | 0.88 | [0.86-0.89] | 33.4 | 0.89 | [0.88-0.90] |
| MELAA[e] | 37.0 | 1.02 | [1.00-1.03] | 37.0 | 1.01 | [1.00-1.03] |
| Other | 37.7 | 1.04 | [0.90-1.20] | 37.7 | 0.99 | [0.86-1.15] |
| **NZDep quintile** | | | | | | |
| 1 (least deprived) | 35.3 | 1.00 | reference | 35.3 | 1.00 | reference |
| 2 | 36.2 | 1.01 | [0.99-1.02] | 36.2 | 1.01 | [1.00-1.03] |
| 3 | 37.3 | 1.02 | [1.01-1.04] | 37.3 | 1.04 | [1.03-1.05] |
| 4 | 38.7 | 1.05 | [1.04-1.06] | 38.7 | 1.07 | [1.06-1.08] |
| 5 (most deprived) | 40.4 | 1.04 | [1.03-1.06] | 40.4 | 1.08 | [1.07-1.09] |
| **BMI category** | | | | | | |
| Underweight (<18) | 34.3 | 0.95 | [0.93-0.98] | 31.4 | 0.94 | [0.92-0.97] |
| Healthy weight (18 - <25) | 36.8 | 1.00 | reference | 34.1 | 1.00 | reference |
| Overweight (25 - <30) | 42.0 | 1.12 | [1.11-1.13] | 39.2 | 1.12 | [1.11-1.13] |
| Obese (≥30) | 47.7 | 1.26 | [1.25-1.27] | 44.9 | 1.26 | [1.25-1.27] |
| **Smoking status** | | | | | | |
| Non-smoker | 39.2 | 1.00 | reference | 37.4 | 1.00 | reference |
| Smoker | 42.6 | 1.07 | [1.06-1.08] | 40.4 | 1.08 | [1.07-1.09] |
| **Parity** | | | | | | |
| Primiparous | 35.1 | 1.00 | reference | 35.0 | 1.00 | reference |
| Non-primiparous | 40.2 | 1.11 | [1.10-1.12] | 39.9 | 1.11 | [1.10-1.12] |

[a] *Denominator includes only those pregnancies that persisted to the start of the trimester*

[b] *Adjusted for all other factors in the table, year of earliest LMP, and clustering by mother*

[c] *Proportions include imputed data (m=40)*

[d] *Ethnicity prioritised according to Statistics New Zealand Level 1 ethnic groups*

[e] *Middle Eastern/Latin American/African*
