# Supplementary material for: Patterns of prescription medicine dispensing before and during pregnancy in New Zealand, 2005–2015
Source: PLoS One. 2020 Jun 2;15(6):e0234153. doi: 10.1371/journal.pone.0234153 (PMC7266349; doi:10.1371/journal.pone.0234153)
Supplement: S9 Table — (PDF) [file pone.0234153.s012.pdf]

S12 Proportions with ≥1 dispensing from Level 2 therapeutic groups; trends before and during pregnancy (with relative risks and 95% confidence intervals)

Pregnancy period	Minerals			Antianaemics			Antibacterials			Analgesics			Antinausea & Antivertigo Agents		
	%	aRR[a]	95% CI	%	aRR[a]	95% CI	%	aRR[a]	95% CI	%	aRR[a]	95% CI	%	aRR[a]	95% CI
Pre-pregnancy 3	3.7	0.20	[0.19-0.20]	2.6	0.10	[0.10-0.10]	13.6	1.23	[1.23-1.24]	7.8	1.17	[1.16-1.18]	1.7	0.21	[0.20-0.21]
Pre-pregnancy 2	3.5	0.19	[0.19-0.19]	3.6	0.14	[0.14-0.14]	14.0	1.27	[1.26-1.28]	8.1	1.21	[1.20-1.22]	1.7	0.21	[0.20-0.21]
Pre-pregnancy 1	3.0	0.16	[0.16-0.16]	4.6	0.18	[0.18-0.18]	13.7	1.25	[1.24-1.26]	7.8	1.17	[1.16-1.18]	1.5	0.19	[0.19-0.19]
Trimester 1	18.7	1.00	reference	25.5	1.00	reference	11.0	1.00	reference	6.7	1.00	reference	8.0	1.00	reference
Trimester 2	20.1	1.06	[1.06-1.07]	4.0	0.16	[0.15-0.16]	14.4	1.31	[1.30-1.32]	9.6	1.43	[1.42-1.45]	4.8	0.59	[0.59-0.60]
Trimester 3	32.1	1.69	[1.68-1.70]	0.8	0.03	[0.03-0.03]	13.1	1.19	[1.18-1.20]	7.9	1.17	[1.16-1.18]	1.5	0.19	[0.19-0.20]

Pregnancy period	Gynaecological Anti-infectives			Corticosteroids Topical			Beta-Adrenoceptor Agonists			Vitamins			Laxatives		
	%	aRR[a]	95% CI	%	aRR[a]	95% CI	%	aRR[a]	95% CI	%	aRR[a]	95% CI	%	aRR[a]	95% CI
Pre-pregnancy 3	1.8	0.74	[0.72-0.75]	3.6	1.15	[1.13-1.16]	3.8	1.10	[1.09-1.12]	1.1	0.48	[0.47-0.49]	1.1	0.75	[0.73-0.77]
Pre-pregnancy 2	1.8	0.74	[0.72-0.75]	3.6	1.14	[2.23-1.16]	3.8	1.12	[1.11-1.13]	1.1	0.48	[0.47-0.49]	1.0	0.71	[0.69-0.73]
Pre-pregnancy 1	1.7	0.69	[0.68-0.71]	3.5	1.12	[3.33-1.13]	3.7	1.10	[1.09-1.11]	1.0	0.45	[0.44-0.46]	0.9	0.62	[0.61-0.64]
Trimester 1	2.4	1.00	reference	3.1	1.00	reference	3.4	1.00	reference	2.3	1.00	reference	1.5	1.00	reference
Trimester 2	4.7	1.95	[1.92-1.99]	4.2	1.35	[5.53-1.37]	4.0	1.18	[1.17-1.20]	2.2	0.97	[0.96-0.99]	2.1	1.45	[1.42-1.49]
Trimester 3	5.5	2.30	[2.26-2.34]	3.4	1.08	[6.63-1.10]	3.7	1.07	[1.06-1.09]	3.7	1.63	[1.60-1.66]	2.4	1.65	[1.61-1.69]

[a] *Adjusted for year of LMP and clustering by mother*

Pregnancy period	Urinary Tract Infections			Antidepressants			Antitrichomonal Agents			Antihistamines			Antiulcerants		
	%	aRR[a]	95% CI	%	aRR[a]	95% CI	%	aRR[a]	95% CI	%	aRR[a]	95% CI	%	aRR[a]	95% CI
Pre-pregnancy 3	1.2	0.73	[0.71-0.75]	4.4	1.41	[1.39-1.43]	1.7	1.12	[1.09-1.14]	2.8	1.41	[1.39-1.44]	1.4	1.21	[1.18-1.24]
Pre-pregnancy 2	1.2	0.73	[0.72-0.75]	4.6	1.45	[1.43-1.47]	1.8	1.18	[1.15-1.20]	2.9	1.47	[1.45-1.50]	1.5	1.24	[1.21-1.27]
Pre-pregnancy 1	1.3	0.75	[0.73-0.77]	4.5	1.42	[1.41-1.44]	1.7	1.15	[1.13-1.18]	2.9	1.49	[1.46-1.51]	1.4	1.21	[1.18-1.24]
Trimester 1	1.7	1.00	reference	3.1	1.00	reference	1.5	1.00	reference	1.9	1.00	reference	1.2	1.00	reference
Trimester 2	2.3	1.39	[1.36-1.42]	2.3	0.74	[0.73-0.75]	2.2	1.44	[1.41-1.48]	1.8	0.89	[0.87-0.91]	1.6	1.33	[1.30-1.36]
Trimester 3	1.7	1.00	[0.97-1.02]	2.2	0.71	[0.70-0.72]	1.4	0.91	[0.88-0.93]	1.5	0.75	[0.73-0.77]	2.7	2.26	[2.21-2.31]

Pregnancy period	NSAIDs			Inhaled Corticosteroids			Local Preparations for Anal & Rectal Disorders			Antacids & Antiflatulants			Nasal Preparations		
	%	aRR[a]	95% CI	%	aRR[a]	95% CI	%	aRR[a]	95% CI	%	aRR[a]	95% CI	%	aRR[a]	95% CI
Pre-pregnancy 3	5.5	2.52	[2.48-2.56]	2.0	1.17	[1.15-1.19]	0.7	1.58	[1.52-1.64]	0.2	0.32	[0.31-0.34]	1.4	1.07	[1.05-1.10]
Pre-pregnancy 2	5.8	2.65	[2.61-2.69]	2.0	1.16	[1.14-1.18]	0.6	1.40	[1.35-1.46]	0.2	0.27	[0.26-0.29]	1.5	1.13	[1.10-1.16]
Pre-pregnancy 1	5.6	2.56	[2.52-2.60]	1.9	1.12	[1.10-1.13]	0.5	1.13	[1.08-1.17]	0.2	0.21	[0.20-0.23]	1.5	1.13	[1.11-1.16]
Trimester 1	2.2	1.00	reference	1.7	1.00	reference	0.4	1.00	reference	0.7	1.00	reference	1.3	1.00	reference
Trimester 2	1.1	0.52	[0.50-0.53]	2.0	1.17	[1.15-1.19]	1.1	2.48	[2.39-2.58]	1.5	2.06	[2.00-2.13]	1.7	1.27	[1.25-1.31]
Trimester 3	0.7	0.32	[0.31-0.34]	1.8	1.04	[1.02-1.06]	2.9	6.45	[6.23-6.68]	2.2	3.11	[3.02-3.21]	1.1	0.83	[0.81-0.86]

Pregnancy period	Contraceptives - Hormonal			Antifungals Topical			Diabetes			Corticosteroids & Related Agents for Systemic Use			Eye Preparations		
	%	aRR[a]	95% CI	%	aRR[a]	95% CI	%	aRR[a]	95% CI	%	aRR[a]	95% CI	%	aRR[a]	95% CI
Pre-pregnancy 3	8.6	4.55	[4.48-4.62]	0.7	1.10	[1.06-1.14]	0.7	0.98	[0.96-1.01]	1.3	1.58	[1.54-1.62]	1.1	1.37	[1.33-1.41]
Pre-pregnancy 2	7.2	3.83	[3.77-3.89]	0.6	1.08	[1.04-1.12]	0.7	1.02	[1.00-1.05]	1.4	1.68	[1.63-1.72]	1.2	1.40	[1.36-1.44]
Pre-pregnancy 1	4.5	2.38	[2.33-2.42]	0.6	0.97	[0.93-1.00]	0.8	1.07	[1.04-1.09]	1.4	1.71	[1.67-1.76]	1.2	1.43	[1.39-1.47]
Trimester 1	1.9	1.00	reference	0.6	1.00	reference	0.7	1.00	reference	0.8	1.00	reference	0.8	1.00	reference
Trimester 2	1.2	0.61	[0.59-0.62]	1.2	1.95	[1.88-2.01]	1.0	1.42	[1.38-1.45]	1.0	1.20	[1.17-1.24]	0.9	1.10	[1.07-1.14]
Trimester 3	0.1	0.06	[0.05-0.06]	1.4	2.26	[2.18-2.34]	2.6	3.68	[3.57-3.80]	0.9	1.05	[1.02-1.09]	0.7	0.81	[0.78-0.84]

[a] *Adjusted for year of LMP and clustering by mother*

Pregnancy period	Antibacterials Topical			Treatments for Substance Dependence			Antithrombotic Agents			Inhaled Long-acting Beta-adrenoceptor Agonists			Sedatives and Hypnotics		
	%	aRR[a]	95% CI	%	aRR[a]	95% CI	%	aRR[a]	95% CI	%	aRR[a]	95% CI	%	aRR[a]	95% CI
Pre-pregnancy 3	0.9	1.31	[1.26-1.35]	0.6	0.77	[0.74-0.79]	0.2	0.27	[0.26-0.28]	1.1	1.01	[1.00-1.03]	1.1	1.75	[1.70-1.80]
Pre-pregnancy 2	0.9	1.36	[1.31-1.40]	0.6	0.81	[0.78-0.84]	0.2	0.28	[0.27-0.30]	1.1	1.05	[1.03-1.06]	1.1	1.85	[1.80-1.91]
Pre-pregnancy 1	0.9	1.31	[1.27-1.36]	0.7	0.85	[0.82-0.87]	0.2	0.30	[0.29-0.31]	1.2	1.06	[1.05-1.08]	1.1	1.77	[1.72-1.82]
Trimester 1	0.7	1.00	reference	0.8	1.00	reference	0.8	1.00	reference	1.1	1.00	reference	0.6	1.00	reference
Trimester 2	0.9	1.23	[1.19-1.27]	0.9	1.07	[1.04-1.10]	1.4	1.67	[1.63-1.71]	1.1	1.02	[1.00-1.04]	0.4	0.60	[0.58-0.63]
Trimester 3	0.7	0.95	[0.92-0.99]	0.5	0.59	[0.57-0.62]	0.8	0.99	[0.96-1.02]	1.1	1.01	[0.99-1.04]	0.5	0.81	[0.78-0.85]

Pregnancy period	Thyroid & Antithyroid Agents		
	%	aRR[a]	95% CI
Pre-pregnancy 3	0.7	0.88	[0.86-0.90]
Pre-pregnancy 2	0.7	0.94	[0.92-0.96]
Pre-pregnancy 1	0.7	1.00	[0.98-1.02]
Trimester 1	0.7	1.00	reference
Trimester 2	0.8	1.08	[1.05-1.10]
Trimester 3	0.8	1.08	[1.05-1.11]

[a] *Adjusted for year of LMP and clustering by mother*
