# Supplementary material for: Patterns of prescription medicine dispensing before and during pregnancy in New Zealand, 2005–2015
Source: PLoS One. 2020 Jun 2;15(6):e0234153. doi: 10.1371/journal.pone.0234153 (PMC7266349; doi:10.1371/journal.pone.0234153)
Supplement: S10 Table — (PDF) [file pone.0234153.s013.pdf]

S13 Proportions with ≥1 dispensing from Level 2 therapeutic groups; trends over study years (with relative risks and 95% confidence intervals)

Therapeutic group	LMP 2005-08		LMP 2009-2011			LMP 2012-2015		
	%		%	RR[a]	95% CI	%	RR[a]	95% CI
Minerals	14.6	ref	39.6	2.70	[2.67 - 2.73]	59.8	4.08	[4.04 - 4.11]
Antianaemics	15.5	ref	29.1	1.87	[1.85 - 1.88]	40.7	2.61	[2.58 - 2.63]
Antibacterials	21.4	ref	28.3	1.31	[1.30 - 1.33]	28.9	1.34	[1.33 - 1.36]
Analgesics	10.8	ref	18.7	1.72	[1.70 - 1.74]	21.6	1.98	[1.96 - 1.01]
Antinausea and Vertigo Agents	6.7	ref	11.3	1.69	[1.66 - 1.71]	15.6	2.31	[2.28 - 2.35]
Gynaecological Anti-infectives	5.8	ref	9.5	1.63	[1.60 - 1.66]	11.0	1.88	[1.85 - 1.92]
Vitamins	2.3	ref	6.4	2.76	[2.69 - 2.84]	9.2	3.97	[3.87 - 3.08]
Corticosteroids Topical	6.2	ref	8.4	1.33	[1.31 - 1.36]	8.4	1.35	[1.32 - 1.37]
Laxatives	2.3	ref	4.5	1.91	[1.86 - 1.96]	6.5	2.76	[2.69 - 2.84]
Beta-Adrenoceptor Agonists	6.0	ref	6.8	1.11	[1.09 - 1.14]	6.3	1.04	[1.02 - 1.06]
Antiulcerants	2.1	ref	3.6	1.70	[1.65 - 1.76]	5.4	2.58	[2.50 - 2.65]
Urinary Tract Infections	3.3	ref	4.7	1.38	[1.35 - 1.42]	5.2	1.53	[1.50 - 1.57]
Antihistamines	2.7	ref	4.3	1.59	[1.55 - 1.64]	5.2	1.90	[1.85 - 1.95]
Antitrichomonal Agents	2.8	ref	4.4	1.56	[1.52 - 1.61]	5.1	1.83	[1.78 - 1.88]
Non-Steroidal Anti-Inflammatory Drugs	2.2	ref	3.8	1.73	[1.68 - 1.79]	4.8	2.18	[2.12 - 2.25]
Antidepressants	3.5	ref	4.4	1.23	[1.20 - 1.26]	4.7	1.31	[1.28 - 1.35]
Local preparations for Anal and Rectal Disorders	1.8	ref	3.3	1.84	[1.78 - 1.90]	4.3	2.39	[2.32 - 2.47]
Nasal Preparations	2.2	ref	3.3	1.51	[1.46 - 1.55]	3.5	1.61	[1.56 - 1.66]
Diabetes	1.3	ref	2.3	1.71	[1.64 - 1.78]	3.3	2.51	[2.42 - 2.61]
Contraceptives - Hormonal	2.5	ref	2.9	1.16	[1.12 - 1.19]	3.2	1.27	[1.23 - 1.31]
Antacids and Antiflatulants	2.2	ref	3.9	1.74	[1.69 - 1.79]	3.1	1.37	[1.33 - 1.42]
Antithrombotic Agents	0.6	ref	1.5	2.68	[2.54 - 2.84]	3.0	5.40	[5.12 - 5.70]
Antifungals Topical	1.7	ref	2.7	1.61	[1.55 - 1.66]	2.7	1.60	[1.54 - 1.66]
Inhaled Corticosteroids	3.6	ref	3.3	0.93	[0.90 - 0.95]	2.5	0.71	[0.69 - 0.73]
Corticosteroids and Related Agents for Systemic Use	1.5	ref	2.2	1.43	[1.38 - 1.48]	2.3	1.53	[1.47 - 1.59]
Treatments for Substance Dependence	0.5	ref	2.3	4.22	[4.00 - 4.44]	2.3	4.22	[4.00 - 4.45]
Eye Preparations	1.6	ref	2.1	1.34	[1.29 - 1.39]	2.2	1.37	[1.32 - 1.42]
Antibacterials Topical	1.3	ref	2.1	1.58	[1.52 - 1.64]	2.0	1.53	[1.47 - 1.59]
Inhaled Long-acting Beta-adrenoceptor Agonists	1.1	ref	1.6	1.38	[1.32 - 1.44]	2.0	1.75	[1.67 - 1.83]
Sedatives and Hypnotics	0.8	ref	1.2	1.49	[1.42 - 1.57]	1.2	1.48	[1.41 - 1.56]
Thyroid and Antithyroid Agents	0.6	ref	1.0	1.53	[1.44 - 1.62]	1.5	2.27	[2.14 - 2.40]

[a] *Adjusted for clustering by mother*
