# Supplementary material for: Patterns of prescription medicine dispensing before and during pregnancy in New Zealand, 2005–2015
Source: PLoS One. 2020 Jun 2;15(6):e0234153. doi: 10.1371/journal.pone.0234153 (PMC7266349; doi:10.1371/journal.pone.0234153)
Supplement: S11 Table — Table includes all therapeutic groups dispensed to at least one cohort member. (PDF) [file pone.0234153.s014.pdf]

## S14 Proportions with ≥1 dispensing from the listed therapeutic groups before and during pregnancy

Table includes all therapeutic groups dispensed to at least one cohort member.

| Therapeutic group[a] | Proportion (%) with ≥1 dispensing from therapeutic group | |
|---|---|---|
| | Whole Pre-pregnancy[b] | Whole pregnancy |
| Minerals | 8.34 | 36.86 |
| Antianaemics | 8.74 | 27.81 |
| Antibacterials | 31.80 | 25.97 |
| Analgesics | 19.08 | 16.71 |
| Antinausea and Vertigo Agents | 4.43 | 10.97 |
| Gynaecological Anti-infectives | 4.66 | 8.65 |
| Corticosteroids Topical | 8.76 | 7.60 |
| Beta-Adrenoceptor Agonists | 7.13 | 6.35 |
| Vitamins | 2.56 | 5.81 |
| Laxatives | 2.73 | 4.36 |
| Urinary Tract Infections | 3.38 | 4.33 |
| Antidepressants | 7.36 | 4.17 |
| Antitrichomonal Agents | 4.74 | 4.05 |
| Antihistamines | 7.21 | 3.99 |
| Antiulcerants | 3.48 | 3.63 |
| Non-Steroidal Anti-Inflammatory Drugs | 14.57 | 3.51 |
| Inhaled Corticosteroids | 3.77 | 3.15 |
| Local preparations for Anal and Rectal Disorders | 1.67 | 3.08 |
| Antacids and Antiflatulants | 0.55 | 3.06 |
| Nasal Preparations | 3.52 | 2.93 |
| Contraceptives - Hormonal | 16.43 | 2.85 |
| Antifungals Topical | 1.76 | 2.34 |
| Diabetes | 1.17 | 2.26 |
| Corticosteroids and Related Agents for Systemic Use | 3.54 | 2.00 |
| Eye Preparations | 3.18 | 1.93 |
| Antibacterials Topical | 2.63 | 1.79 |
| Treatments for Substance Dependence | 1.61 | 1.63 |
| Antithrombotic Agents | 0.47 | 1.63 |
| Inhaled Long-acting Beta-adrenoceptor Agonists | 1.68 | 1.53 |
| Sedatives and Hypnotics | 2.43 | 1.07 |
| Thyroid and Antithyroid Agents | 1.01 | 1.02 |
| Parasiticidal Preparations | 1.18 | 0.90 |
| Antivirals | 1.15 | 0.82 |
| Beta Adrenoceptor Blockers | 0.68 | 0.79 |
| Urinary Agents | 1.21 | 0.78 |
| Anxiolytics | 1.50 | 0.63 |
| Centrally-Acting Agents | 0.13 | 0.57 |
| Antispasmodics and Other Agents Altering Gut Motility | 1.42 | 0.54 |
| Antipsychotics | 0.79 | 0.53 |

| | | |
|---|---|---|
| Antimigraine Preparations | 1.32 | 0.53 |
| Other Endocrine Agents | 1.36 | 0.51 |
| Mouth and Throat | 0.84 | 0.48 |
| Ear/Eye Preparations | 0.78 | 0.43 |
| Antidiarrhoeals | 1.06 | 0.43 |
| Antiepilepsy Drugs | 0.60 | 0.41 |
| Hormone Replacement Therapy - Systemic | 1.19 | 0.41 |
| Scalp Preparations | 0.76 | 0.39 |
| Ear Preparations | 0.55 | 0.39 |
| Antifungals | 1.18 | 0.28 |
| Antiandrogen Oral Contraceptives | 1.67 | 0.23 |
| Antifibrinolytics, Haemostatics and Local Sclerosants | 0.79 | 0.22 |
| Agents Affecting the Renin-Angiotensin System | 0.53 | 0.21 |
| Calcium Channel Blockers | 0.23 | 0.21 |
| Other Progestogen Preparations | 0.59 | 0.19 |
| Psoriasis and Eczema Preparations | 0.32 | 0.16 |
| Lipid-Modifying Agents | 0.27 | 0.12 |
| Anticholinergic Agents | 0.17 | 0.12 |
| Diuretics | 0.29 | 0.10 |
| Muscle Relaxants | 0.46 | 0.10 |
| Immunosuppressants | 0.13 | 0.09 |
| Antiacne Preparations | 0.42 | 0.09 |
| Digestives Including Enzymes | 0.01 | 0.08 |
| Stimulants/ADHD Treatments | 0.08 | 0.06 |
| Antirheumatoid Agents | 0.09 | 0.05 |
| Wart Preparations | 0.18 | 0.05 |
| Antiarrhythmics | 0.04 | 0.04 |
| Agents for Parkinsonism and Related Disorders | 0.14 | 0.04 |
| Anthelmintics | 0.10 | 0.04 |
| Extemporaneously Compounded Preparations and Galenicals | 0.05 | 0.03 |
| Hyperuricaemia and Antigout | 0.10 | 0.03 |
| Chemotherapeutic Agents | 0.06 | 0.03 |
| Alpha Adrenoceptor Blockers | 0.05 | 0.03 |
| Anaesthetics | 0.02 | 0.02 |
| Mast Cell Stabilisers | 0.03 | 0.02 |
| Myometrial and Vaginal Hormone Preparations | 0.15 | 0.02 |
| Nitrates | 0.02 | 0.02 |
| Antiretrovirals | 0.01 | 0.01 |
| Endocrine Therapy | 0.04 | 0.01 |
| Antituberculotics and Antileprotics | 0.04 | 0.01 |
| Drugs Affecting Bone Metabolism | 0.02 | 0.01 |
| Sex Hormones Non Contraceptive | 0.04 | 0.01 |
| Sympathomimetics | 0.01 | 0.01 |
| Vasopressin Agonists | 0.01 | 0.01 |
| Trophic Hormones | 0.06 | 0.01 |
| Methylxanthines | 0.01 | 0.01 |
| Antiparasitics | 0.02 | 0.01 |
| Leukotriene Receptor Antagonists | 0.01 | 0.00 |
| Anticholinesterases | 0.00 | 0.00 |

| | | |
|---|---|---|
| Multiple Sclerosis Treatments | 0.01 | 0.00 |
| Agents Used in the Treatment of Poisonings | 0.00 | 0.00 |
| Other Oestrogen Preparations | 0.02 | 0.00 |
| Mucolytics | 0.00 | 0.00 |
| Immune Modulators | 0.00 | 0.00 |
| Vasodilators | 0.00 | 0.00 |
| Topical Products for Joint and Muscular Pain | 0.00 | 0.00 |
| Antihypotensives | 0.00 | 0.00 |
| Blood Colony-stimulating Factors | 0.00 | 0.00 |
| Treatments for Dementia | 0.00 | 0.00 |
| Cough Preparations | 0.00 | 0.00 |
| Respiratory Stimulants | 0.00 | 0.00 |

[a] *PHARMS Level 2 therapeutic groups*

[b] *1-270 days pre-conception*
