# Supplementary material for: Patterns of prescription medicine dispensing before and during pregnancy in New Zealand, 2005–2015
Source: PLoS One. 2020 Jun 2;15(6):e0234153. doi: 10.1371/journal.pone.0234153 (PMC7266349; doi:10.1371/journal.pone.0234153)
Supplement: S12 Table — (PDF) [file pone.0234153.s015.pdf]

S15 Proportions with ≥1 dispensing from Level 2 therapeutic groups during Trimester 1, by grouped pregnancy outcome

Pregnancy outcome	Analgesics			Antacids & Antiflatulants			Antianaemics			Antibacterials		
	%	aRR[a]	95% CI	%	aRR[a]	95% CI	%	aRR[a]	95% CI	%	aRR[a]	95% CI
Deliveries	6.7	1.00	reference	0.9	1.00	reference	31.5	1.00	reference	10.6	1.00	reference
Non-deliveries[b]	7.5	1.12	[1.09-1.14]	0.3	0.35	[0.31-0.38]	14.1	0.45	[0.45-0.46]	13.8	1.30	[1.28-1.32]
Undetermined[c]	6.4	0.98	[0.95-1.00]	0.3	0.32	[0.29-0.36]	13.9	0.46	[0.45-0.46]	10.8	1.03	[1.01-1.04]

Pregnancy outcome	Antibacterials Topical			Antidepressants			Antifungals Topical			Antihistamines		
	%	aRR[a]	95% CI	%	aRR[a]	95% CI	%	aRR[a]	95% CI	%	aRR[a]	95% CI
Deliveries	0.7	1.00	reference	2.6	1.00	reference	0.6	1.00	reference	1.7	1.00	reference
Non-deliveries[b]	0.8	1.16	[1.08-1.24]	5.1	1.95	[1.89-2.01]	0.5	0.83	[0.76-0.90]	2.3	1.30	[1.25-1.35]
Undetermined[c]	0.7	1.13	[1.06-1.20]	3.7	1.46	[1.41-1.50]	0.6	0.96	[0.89-1.03]	2.6	1.53	[1.48-1.59]

Pregnancy outcome	Antinausea & Antivertigo Agents			Antithrombotic Agents			Antitrichomonal Agents			Antiulcerants		
	%	aRR[a]	95% CI	%	aRR[a]	95% CI	%	aRR[a]	95% CI	%	aRR[a]	95% CI
Deliveries	9.7	1.00	reference	1.0	1.00	reference	1.2	1.00	reference	1.1	1.00	reference
Non-deliveries[b]	5.7	0.59	[0.57-0.60]	0.6	0.58	[0.53-0.63]	3.3	2.83	[2.73-2.94]	1.2	1.06	[1.00-1.12]
Undetermined[c]	3.8	0.40	[0.39-.041]	0.3	0.28	[0.25-0.31]	1.5	1.28	[1.22-1.34]	1.5	1.31	[1.25-1.38]

[a] *Adjusted for year of LMP and clustering by mother*

[b] *Includes deliveries of live and stillborn infants*

[c] *Includes terminations, miscarriages, other early pregnancy losses*

Pregnancy outcome	Beta-Adrenoceptor Agonists			Contraceptives - Hormonal			Corticosteroids & Related Agents for Systemic Use			Corticosteroids Topical		
	%	aRR[a]	95% CI	%	aRR[a]	95% CI	%	aRR[a]	95% CI	%	aRR[a]	95% CI
Deliveries	3.4	1.00	reference	0.8	1.00	reference	0.7	1.00	reference	3.1	1.00	reference
Non-deliveries[b]	4.0	1.16	[1.13-1.20]	4.5	5.31	[5.11-5.51]	1.2	1.66	[1.57-1.76]	3.3	1.05	[1.02-1.09]
Undetermined[c]	3.1	0.92	[0.89-0.95]	3.7	4.38	[4.22-4.55]	1.0	1.43	[1.35-1.52]	3.2	1.05	[1.02-1.08]

Pregnancy outcome	Diabetes			Eye Preparations			Gynaecological Anti-infectives			Inhaled Corticosteroids		
	%	aRR[a]	95% CI	%	aRR[a]	95% CI	%	aRR[a]	95% CI	%	aRR[a]	95% CI
Deliveries	0.7	1.00	reference	0.8	1.00	reference	2.5	1.00	reference	1.7	1.00	reference
Non-deliveries[b]	0.6	0.89	[0.83-0.97]	0.9	1.12	[1.05-1.20]	2.6	1.05	[1.01-1.09]	1.9	1.08	[1.03-1.13]
Undetermined[c]	0.9	1.40	[1.32-1.49]	1.1	1.38	[1.31-1.46]	1.9	0.76	[0.73-0.79]	1.6	0.93	[0.89-0.98]

Pregnancy outcome	Inhaled Long-acting Beta Agonists			Laxatives			Local preparations for Anal and Rectal Disorders			Minerals		
	%	aRR[a]	95% CI	%	aRR[a]	95% CI	%	aRR[a]	95% CI	%	aRR[a]	95% CI
Deliveries	1.1	1.00	reference	1.7	1.00	reference	0.5	1.00	reference	23.8	1.00	reference
Non-deliveries[b]	1.2	1.12	[1.06-1.18]	1.1	0.62	[0.59-0.66]	0.4	0.78	[0.71-0.86]	10.2	0.43	[0.43-0.44]
Undetermined[c]	1.1	1.01	[0.95-1.07]	0.8	0.48	[0.46-0.51]	0.4	0.94	[0.87-1.03]	7.9	0.35	[0.35-0.36]

[a] Adjusted for year of LMP and clustering by mother

[b] Includes deliveries of live and stillborn infants

[c] Includes terminations, miscarriages, other early pregnancy losses

Pregnancy outcome	Nasal Preparations			Non-Steroidal Anti-Inflammatory Drugs			Sedatives & Hypnotics			Thyroid and Antithyroid Agents		
	%	aRR[a]	95% CI	%	aRR[a]	95% CI	%	aRR[a]	95% CI	%	aRR[a]	95% CI
Deliveries	1.3	1.00	reference	0.3	1.00	reference	0.4	1.00	reference	0.8	1.00	reference
Non-deliveries[b]	1.2	0.89	[0.84-0.94]	0.3	1.09	[0.97-1.23]	1.2	3.08	[2.89-3.29]	0.7	0.88	[0.82-0.95]
Undetermined[c]	1.5	1.18	[1.12-1.23]	0.4	1.51	[1.37-1.67]	1.0	2.54	[2.38-2.72]	0.8	1.10	[1.03-1.18]

Pregnancy outcome	Treatments for Substance Dependence			Urinary Tract Infections			Vitamins		
	%	aRR[a]	95% CI	%	aRR[a]	95% CI	%	aRR[a]	95% CI
Deliveries	0.8	1.00	reference	1.8	1.00	reference	2.7	1.00	reference
Non-deliveries[b]	0.9	1.06	[0.99-1.13]	1.5	0.83	[0.79-0.87]	1.3	0.48	[0.46-0.51]
Undetermined[c]	0.5	0.68	[0.63-0.73]	1.4	0.77	[0.74-0.81]	1.5	0.57	[0.54-0.59]

[a] *Adjusted for year of LMP and clustering by mother*

[b] *Includes deliveries of live and stillborn infants*

[c] *Includes terminations, miscarriages, other early pregnancy losses*
